# The Impact of Soil-Applied Biochars From Different Vegetal Feedstocks on Durum Wheat Plant Performance and Rhizospheric Bacterial Microbiota in Low Metal-Contaminated Soil

**DOI:** 10.3389/fmicb.2019.02694

**Published:** 2019-12-10

**Authors:** Arianna Latini, Giovanni Bacci, Manuel Teodoro, Daniele Mirabile Gattia, Annamaria Bevivino, Lukáš Trakal

**Affiliations:** ^1^Italian National Agency for New Technologies, Energy and Sustainable Economic Development, ENEA Casaccia Research Centr, Rome, Italy; ^2^Department of Biology, University of Florence, Florence, Italy; ^3^Department of Environmental Geosciences, Faculty of Environmental Sciences, Czech University of Life Sciences Prague, Prague, Czechia

**Keywords:** biochar, durum wheat, vegetal feedstock, rhizosphere bacterial microbiome, low-metal contaminated soil

## Abstract

Biochar shapes the soil environment and plant growth. Nevertheless, the mechanisms associated with an improved plant biomass and soil microbiome in low metal-contaminated soils are still unclear. In this study, the influence of biochar on soil physico-chemical properties, plant performance, and rhizosphere microbiota in durum wheat was investigated at the above- and belowground levels. Two kinds of biochar from different feedstocks (wood chips and wheat straw pellets) and two Italian durum wheat varieties, Duilio and Marco Aurelio, were analyzed in a greenhouse using a low-nutrient gleyic fluvisol containing a very small amount of Pb and Zn. Four different treatments were performed: soil-only control (C), soil amended with woody biochar equilibrated with nutrient solution (B1+) and non-activated (B1−), and soil amended with non-activated (B2−) wheat straw biochar. Seven weeks after seed germination, (1) the physico-chemical properties of soil, biochars, and mixtures were assessed; (2) the fresh and dry weight of aboveground plant tissues and roots and other morphometric traits were measured; and (3) metabarcoding of the 16S rRNA bacterial gene was performed on rhizosphere soil samples. The results showed that the biochar from wheat straw had stronger impact on both durum varieties, with higher electrical conductivity, higher levels of available K and Na, and a substantial increase of dissolved Na+, K+, and Cl− ions in pore water. Generally, biochar amendment decreased Zn availability for the plants. In addition, biochar improved plant growth in the early growth stage, and the more positive effect was achieved by combining wheat straw biochar with Marco Aurelio. Rhizosphere bacterial microbiota showed variation in alpha diversity only due to treatment; on the other hand, the differential analysis showed consistent variation among samples with significant effects on amplicon sequence variant (ASV) abundance due to the specific biochar treatment as well as the genotype. The pure B1−, due to its scarce nutrient content with respect to the richer types (B1+ and B2−), had a negative impact on microbiota richness. Our study highlights that an appropriate combination of biochar feedstock and crop species may lead to superior yield.

## Introduction

In the last decade, biochar has been focused upon due to its great potential for climate change mitigation, and its application to soil has emerged as an attractive strategy for sequestering carbon, reducing greenhouse gas (GHG) emissions, and improving soil quality (Lehmann, 2007; Atkinson et al., 2010; Agegnehu et al., 2016). Biochar may have variable effects on (i) soil properties, (ii) soil biota, including microbiota (Lehmann et al., 2011), (iii) plant growth and crop yield (Biederman and Harpole, 2013; Jefferey et al., 2015; Pandit et al., 2018; Sun et al., 2019), (iv) roots (Brennan et al., 2014; Prendergast-Miller et al., 2014; Xiang et al., 2017) and the rhizosphere microbiome (De Tender et al., 2016; Kolton et al., 2017), and (v) crop resistance to disease (Elad et al., 2011; Frenkel et al., 2017). Biochar has also been proved to be effective in the remediation of soils with both heavy metal and organic pollutants (Brennan et al., 2014; Zama et al., 2018), playing a critical role in reducing ecological and human health risks associated with heavy metal contamination.

The structure and function of biological communities within soils are complex, and the presence and variable abundance of individual members have a profound effect on soil function, plant health, and productivity (Atkinson et al., 2010). Several studies have reported that soil microorganisms are affected following biochar application, with it increasing or decreasing (Han et al., 2017; Kolton et al., 2017; Li et al., 2018) their biomass, while others have found that amendment with biochar had no significant effect (Lehmann et al., 2011). These variable microbial responses are controlled by multiple environmental factors like types and rates of biochar amendment, the initial edaphic conditions, land use and management regimes, and vegetation types. The results of next-generation sequencing across Europe have indicated that biochar has significant effects on soil microbial communities, even if these may be small with respect to the high microbiome variability in different soils (Jenkins et al., 2017). Very little is known about the mechanisms through which different types of biochar in the same soil environment affect the microbial abundance and community composition, and this has recently been reviewed by Palansooriya et al. (2019). Concerning microbial abundance and activities, pH is one of the soil chemical properties that have a major influence (Lehmann et al., 2011).

A comprehensive understanding of how soil microbial communities respond to different biochar amendments, taking into account the effects of different biochar feedstocks, pyrolysis protocols, concentrations in the soil, and so on, is still far off. Also, the cultivar-specific response of the bacterial community to soil-applied biochars in the rhizosphere of certain plant species needs to be further investigated. Choosing the best combination of cultivar and soil treatment could amplify the benefit of biochar-based practices and increase agricultural sustainability.

Durum wheat (*Triticum turgidum* L. subsp. *durum*), even though representing only 5% of total wheat, is an economically important crop due to its unique characteristics and end products, in particular pasta. It is better adapted to semiarid climates than bread wheat. Only a few experiments are reported in the literature that focus on durum wheat performance in the presence of biochar. Among the more noteworthy results on this topic, durum wheat showed a 10% increase in grain production after a biochar application of 10 t ha^–1^ in the field in Central Italy (Baronti et al., 2010). Vaccari et al. (2011) applied a large volume of biochar (30 and 60 t ha^–1^) to the soil for durum wheat planting in the Mediterranean climate condition and obtained an increase of up to 30% on biomass production and yield. At the same time, they also evaluated the overall impact of biochar on soil microbial activity, which reached a minimum over the first 14 months after biochar incorporation (Castaldi et al., 2011). Moreover, biochar addition to a nutrient-poor, slightly acidic loamy sand pot soil had little effect on durum wheat yield in the absence of mineral fertilization, while it produced a 20–30% increase in grain yield at the highest mineralization rate (Albuquerque et al., 2013). The same team of researchers reported that durum wheat treated with biochar from olive-tree pruning in the field in the Mediterranean showed higher relative growth, aboveground biomass, and yield than control plants, in accordance with Vaccari et al. (2011). They also demonstrated that plants responded to biochar addition by increasing fine root proliferation (Olmo et al., 2014). Furthermore, the soil microbial community structure has been analyzed in a 2-year durum wheat field trial in Italy under two loads of woody biochar (30 and 60 t ha^–1^), without any apparent effect on microbial biomass, activity, or diversity (Rutigliano et al., 2014).

Until now, the effect of soil-applied biochars from different stock biomasses on rhizosphere microbiota, durum wheat plant performance, and soil properties in low metal-contaminated soil has not been investigated. This study focused on soil originating from the vicinity of a mining/smelting district (Přibram, Czechia), 12 km distant from the heavily contaminated soil, and followed up several previous studies dealing with bulk metal contamination in this area (Zemanová et al., 2014; Jačka et al., 2018). In the present study, two different biochars (i.e., wood and wheat straw), both manufactured at a high pyrolysis temperature (700°C), and two durum wheat varieties exhibiting different behavior and traits, thus reflecting the level of influence of the plant genotype to the biochar treatment, were used with the aim of assessing the effect of biochar type and/or durum genotype on the diversity and composition of microbiota associated with rhizosphere soil, soil properties, and plant growth. This study was designed to establish the relationship of biochar-induced changes in rhizosphere bacterial community structure with soil nutrient composition and the growth promotion of durum wheat in a comprehensive fashion.

## Materials and Methods

### Soil and Biochars

The soil used originated from a gleyic fluvisol coming from the alluvium of the Litavka river (Láz close to the Přibram mining/smelting district, Czechia). It had 2.2% SOM and a negligible amount of Pb and Zn, with a total content of both metals of up to 100 ppm; it is classified as sandy loam soil according to U.S.D.A. taxonomy. Soil samples were collected from the arable layer (0–25 cm), air-dried, sieved through a 2-mm stainless sieve, and homogenized. The soil texture was 8.7% clay (<2 μm), 34.8% silt (2–50 μm), and 56.5% sand (0.05–2 mm); the bulk density (ρ) was 1.21 g cm^–3^, and the soil porosity was 0.58, as presented in Jačka et al. (2018).

Two types of biochars produced from different feedstocks were used for soil amendment; i.e., wood chip biochar (referred in this manuscript as B1) provided by Carbon Terra (Germany), and wheat straw pellet biochar (WSP700, referred here as B2) provided by the UK Biochar Research Center (2014) (University of Edinburgh, United Kingdom). Both biochars were produced at a high pyrolysis temperature of 700°C, as reported in Supplementary Table S1 in Supplementary Material S1 together with their other characteristics. These biochars are considered as standard; in fact, they have been widely investigated by several research teams (Wiedner et al., 2013; Kammann et al., 2015; Shen et al., 2017a, b; Kaetzl et al., 2018; Mašek et al., 2018; and several others) and were also used in this study for experimental reproducibility purposes.

Both untreated (B1−) and activated (B1+) woody biochars were employed; in this second form, biochar underwent conditioning incubation in full-nutrient Hoagland solution (Hoagland and Arnon, 1950) as fertilizer for 1 week. As the wheat straw biochar (B2) contained a sufficient quantity of nutrients (e.g., N, see Supplementary Table S1 in Supplementary Material S1), activation by Hoagland solution was not performed for this case, and only the untreated (B2−) form was used.

Both soil and biochars were air-dried and finely ground (>2 mm) before soil mixture preparation. For samples different from controls, soil was mixed with the respective biochar (either as-such or previously activated) at a 3% *w/w* dosage.

### Plants

Two Italian high-yielding durum wheat (*Triticum turgidum* L. subsp. *durum*) varieties, Duilio (V1) and Marco Aurelio (V2), chosen from among a panel of Italian elite genotypes previously analyzed^1^ (unpublished results), were used. Their morpho-physiological characteristics, their tolerant/resistant behavior with respect to major biotic and abiotic stresses, and the main qualitative information regarding them are reported in Supplementary Table S2 in Supplementary Material S2. These two varieties were chosen due to their different wheat biomass production in the early growth stage upon biochar addition: Duilio showed a positive effect on shoot biomass production in the presence of biochar from wood feedstock, while Marco Aurelio did not show improved performance in the presence of biochar from either wood or wheat straw feedstock (unpublished data).

Plant seeds were kindly provided by the Società Italiana Sementi (SIS), which describes Duilio as a good-yielding variety in most kinds of soil, an early and highly rustic wheat, very widespread, and reports for Marco Aurelio an excellent productivity, high protein content, high yellow index in semolina, wide adaptability, and tolerance to Septoria.

### Greenhouse Experimental Design

A pot trial was carried out under greenhouse conditions in Prague at the Czech University of Life Science in March 2017. The climatic conditions set in the greenhouse during this experiment were: 24°C/18°C day/night temperature, 70% relative humidity, and a 16 h photoperiod. The experiment was laid out in a complete randomized block design with nine replicates in polyvinyl chloride (PVC) pots (12-cm top diameter, 10-cm bottom diameter, 10-cm height, with a volume of 1 L). Treatments were as follows: (i) soil-only control (C), (ii) soil plus untreated woody biochar (B1-), (iii) soil plus activated woody biochar (B1+), and (iv) soil plus untreated wheat straw biochar (B2−). This led to an experimental size of eight samples (2 plant varieties × 4 treatments), each of them with nine biological replicates, thus resulting in a total of 72 plants (1 plant/pot). Both soil and biochars were air-dried and finely ground (>2 mm) before soil mixture preparation. For samples different from controls, soil was mixed with the respective biochar (either as-such or previously activated) at a 3% *w/w* dosage. No further fertilization was applied to plants.

Before planting, seeds were surface-sterilized in 5% NaOCl for 2 min, then rinsed in three changes of sterile distilled water. Afterward, disinfected seeds were germinated onto filter paper (Whatman 1), moistened with 10 ml of sterile distilled water, in 100 × 10 mm Petri dishes. After 6 days, plantlets were transplanted into pots. In the beginning, three plantlets per pot were sown at a depth of 1 cm; then, after 10–12 days when most of the plants had emerged, only one plant was chosen and preserved. After planting, 100 g of the washed inert silica sand (>2 mm) was placed over the soil of each pot to minimize water evaporation.

Initial irrigation was realized gravitationally (using the counted weight of demi-water) in order to reach the given value of WHC = 60% (for a soil porosity of 0.58). Afterward, plants received irrigation three times per week by carefully pouring of deionized water onto the surface of the potting soil with a graduated cylinder. In each irrigation, each pot was weighed on a top-loading balance before watering to calculate the amount of water to be supplied. Considering that the pot and saucer weight was around 75 g, that each pot was filled with 900 g of dried soil or soil-biochar mixture, and that the approximate weight of water at 60% WHC was 300 g, varying slightly according to the particular soil treatment and the plant growth, the total pot weight was around 1,275 g (75 + 900 + 300 g), excluding the water-filled tube (explained hereinafter). Moreover, to ensure constant moisture (60% of WHC) from one watering time to another throughout the entire experiment duration, 15-ml Falcon tubes equipped with irrigation wicks were placed into the soil, with the volume of water in the tubes being regularly restored by filling (Supplementary Material S3).

Plants were checked regularly, and their phenological phases were assessed on the Feekes scale throughout the experiment. Six weeks after transplanting (final time, T_f_), plants had grown enough for further analyses. First, bulk soil and soil-biochar mixtures were collected from all of the pots for their chemical-physical characterization at the final time of the experiment (T_f_). In this case, pot soil samples were kept separated for the two plant varieties. Moreover, before harvesting (T_f_), soil pore water was collected in each pot with 10-cm long rhizones (Eijkelkamp, Netherlands). Second, plant aboveground and belowground biomasses at T_f_ were evaluated for all 72 plants (9 replicates/condition) through their FW and DW measurement in order to estimate plant growth. Third, six biological replicates, randomly chosen out of the nine at the beginning of the experiment, were used for rhizosphere sampling for metagenomics analyses.

### Biochar Microstructure Analysis

In order to ascertain the microstructure of the two biochars used, Scanning Electron Microscopy (SEM) has been performed with a Zeiss EVO MA15 operated at 20 kV. The samples were directly observed after supporting them on an aluminum stab covered with conductive carbon tape. X-ray diffraction was used in order to study the presence of crystalline phases in the biochar samples. A Rigaku SmartLab powder diffractometer, equipped with a monochromator in the diffracted beam and a Cu Kα radiation source (λ = 1.5405 Å), was operated at 40 kV and 30 mA in the range of 10–90 2θ, with a step size of 0.04, and 8 s per step. The biochars were reduced to a fine powder using an agate mortar and pestle.

### Soil and Soil-Biochar Mixture Analysis

At time zero of the experiment (T_0_) as well as at the end (T_f_), the soil replicas for each variant were mixed together (V1C, V1B1−, V1B1+, and V1B2−; V2C, V2B1−, V2B1+, and V2B2−) and analyzed per treatment and per genotype in duplicate. Each soil sample was air dried, homogenized, and again sieved (<2 mm) to remove any residue of silica sand or plant roots. Determination of pH was measured in distilled water and a KCl suspension at a 1:5 (*w*/*v*) ratio (according to the ISO 10390:2005 standard for soil quality) using a pH meter (inoL^a^b^®^ pH 7310, WTW, Germany). Electric Conductivity (EC) was obtained from a 1:5 (*w*/*v*) H_2_O suspension (USDA Soil Survey Staff, 2014) using a Multi 3420 (WTW, Germany) digital precision meter. CEC was determined using the 0.1 M BaCl_2_ (1:5*0 w*/*v*) protocol (Carter and Gregorich, 2008). Total organic/inorganic C in soil was determined using a SSM-5000A (Shimadzu, Japan) carbon analyzer.

For the directly available metal pool, samples of 2 g of soil were treated with 20 ml of 0.01 M CaCl_2_ (Quevauviller, 1998), shaken for 3 h at 300 rpm, centrifuged for 10 min at 3000 rpm, and filtered through a 0.45 μm nylon filter (VWR, Germany). Pseudo total concentrations of elements were extracted by adding 10 ml of *aqua regia* (2.5 ml HCl and 7.5 ml HNO_3_) to 0.5 g of dry soil and were digested at 200°C under microwave conditions (SPD-Discover, CEM, United States). The samples were diluted in 25 ml of deionized water and filtered through a 0.45 μm nylon filter. The concentrations of elements in the solutions obtained were analyzed by inductively coupled plasma optical emission spectrometry (ICP-OES; 720ES, Varian Inc., Palo Alto, CA, United States). The standard reference materials 2710a Montana Soil I (NIST, United States) and CRM 483 (Institute for Reference Materials and Measurements, EU) were used.

Determinations of pH and EC in pore water were performed using standard equipment, as reported above. Major inorganic anions + cations were determined using a Dionex ICS-5000 ion chromatography system (Dionex, United States) and inductively coupled plasma optical emission spectrometry (ICP-OES).

### Evaluation of Plant Performance in the Early Growth Stage

Fresh (FW) and dry weights (DW) were measured on an analytical balance at T_f_. The aboveground part of each plant was kept separate from the roots. Before measuring the FW of the roots, they were washed in water and laid on filter paper to remove excess water. The values of DW of roots were recorded after 2 days of incubation at 75°C. At T_f_, morphometric traits of the plants, namely the PH from the base of the stem up to the end of the emerging spike, FLL, and maximum width (FLMW), were measured in *cm*. Lastly, the number of plant leaves was also noted.

### Rhizosphere Sampling for Metagenomics Analysis

After the removal of the silica sand on the soil surface with a spatula, pot plants (2 varieties × 4 treatments × 6 replicates, for a total of 48 plants) were turned upside down on filter paper. Each plant was carefully removed from the bulk soil and shaken vigorously to remove loosely adhered soil particles. Roots with adhering soil were covered in aluminum foil; then, in the lab, the plant was dissected. The entire root (belowground tissue) containing tightly adhering soil was put in a 15-ml Falcon tube containing 10 ml autoclaved 0.9% (*w*/*v*) sodium chloride (NaCl) solution and then shaken at 50 rpm for 20 min at room temperature in a multirotator (multiRS-biosan) to free root-associated bacterial cells. Root tissue samples were then removed from the suspension, samples were centrifuged at 6,500 × *g* for 15 min, and the supernatant was decanted. The derived pellet was highly enriched in root-associated bacteria and stored at −80°C until DNA extraction.

### DNA Extraction and PCR Amplification

DNA was extracted from 500 mg rhizosphere soil samples using a FastDNA^®^ SPIN Kit for Soil in combination with a FastPrep-24^TM^ 5G homogenizer (MP Biomedicals), according to the manufacturer’s instructions. In order to optimize quality and A_230_ value, the extracted DNA was further diluted and concentrated by VIVASPIN 500 centrifugal concentrators (10,000 MWCO).

The extracted soil gDNA was run on a 1.0% agarose gel and quantified using both a Nanodrop ND-1000 spectrophotometer (NanoDrop Technologies, Wilmington, DE, United States) and a Qubit 4 Fluorometer (Invitrogen by Thermo Fisher Scientific Inc.) (Supplementary Material S4) for quality-quantity check. A PCR test with primers P0 and P6 for amplification and sequencing of bacterial 16S rDNA (Di Cello et al., 1997) was also performed on a few randomly chosen DNA samples.

For each of the 48 collected rhizosphere samples, we performed two independent DNA extractions, and then we pooled them in an equimolar ratio, obtaining a composite root-associated DNA sample for each plant.

### Illumina 16S Library Construction and Sequencing

The DNA concentration of the samples was adjusted to 10 ng/μl and then diluted at 1:20 for the subsequent investigations. The sequencing protocol was performed at BMR Genomics Srl (Padua, Italy). Briefly, the V3-V4 regions of 16S rRNA gene were amplified using the following primers: Pro341F, 5′-CCTACGGGNBGCASCAG-3′, and Pro805R, 5′-GACTACN VGGGTATCTAATCC-3′ (Takahashi et al., 2014). Primers were modified with the forward and reverse overhangs (5′-TCGT CGGCAGCGTCAGATGTGTATAAGAGACAG-[locus-specific sequence]-3′ and 5′-GTCTCGTGGGCTCGGAGATGTGTAT AAGAGACAG-[locus-specific sequence]-3′, respectively) necessary for dual index library preparation. Amplicons were purified by 0.8x Agencourt AMPure XP magnetic beads (Beckman Coulter) and amplified with a short cycle with a Nextera XT Index (Illumina). They were then normalized by SequalPrep (Thermo Fisher) and multiplexed. The pool was purified by 1x Agencourt AMPure XP magnetic beads (Beckman Coulter), loaded on Illumina Miseq, and sequenced with a 300PE v3 chemistry strategy.

### Amplicon Sequence Variant Inference

Sequences were clustered into ASVs using the DADA2 pipeline outlined at *https://benjjneb.github.io/dada2/tutorial.html* (Callahan et al., 2016). Before running the pipeline, PCR primers were removed with cutadapt (Martin, 2011) using the default settings. Sequences that were not trimmed by the software (namely those where the adapter was not found) were removed from the analysis (–discard-untrimmed option). If only one read of a pair was removed, the other one was also discarded to maintain the paired-end nature of the samples (–pair-filter = any option). Sequences were then filtered using the filterAndTrim function of DADA2 with a maximum error rate of 2. The truncLen option was set to 270 for forward reads and 200 for reverse reads in order to maintain more than 20 bp of overlap while removing low-quality tails. Trimmed sequences were used for error rate estimation (the learnErrors function with default parameters). Finally, sequences were denoised and merged, and variants were inferred using the DADA2 algorithm. Taxonomic annotation was carried out after chimera removal using the Silva training set 128 (Quast et al., 2012). The number of sequences retained in every step is reported in Supplementary Material S5, together with the resulting rarefaction analysis. Samples with a final number of reads lower than 10,000 were removed from subsequent analyses (five samples in total). Consistent ASVs were detected by comparing three technical replicates, as described in Supplementary Material S6, which provides additional information on the processing.

### Differential Analysis and Taxonomic Distribution

Differential abundance analysis was performed on consistent ASVs using DESeq2 (Love et al., 2014). Fold changes were shrunk using the adaptive shrinkage estimator from the ‘ashr’ package (Stephens, 2016). All *p*-values were adjusted using the Benjamini and Hochberg correction (also known as the “false discovery rate”), and only contrasts reporting a *p*-value lower than 0.05 with an absolute log2-fold-change value higher than 1 have been considered.

For the taxonomic distribution of consistent ASVs, samples and taxa were clustered using the unweighted pair group method with arithmetic mean (UPGMA) based on the Bray–Curtis distance.

### Statistical Analysis

Plant data were statistically analyzed by one-way ANOVA (with Tukey HSD *post hoc* test) with IBM SPSS Statistics 23 software, setting *p* < 0.05 as the significance level. The mean FW and DW values (in grams) proceeding from nine replicated samples were treated as the dependent variables, while the treatments and the two genotypes (V1C, V1B1−, V1B1+, and V1B2−; V2C, V2B1−, V2B1+, and V2B2−) as the independent variables. Before conducting one-way ANOVA, the normal distribution of the data was checked by Shapiro–Wilk test (Sig. > 0.05), and the homogeneity of variances was checked by Levene test (Sig. > 0.05; Supplementary Materials S7, S8).

Linear regression was carried out to inspect the relation between the abundance and persistence of bacterial ASVs, whereas one-way analysis of variance (ANOVA) was performed on alpha diversity to inspect the effect of genotype and treatment. Alpha diversity values were inspected without applying any transformation, whereas abundance data were log-transformed before analysis. Consistent taxa (and samples) were clustered using the unweighted pair group method with arithmetic mean (UPGMA) based on the Bray–Curtis distance. Differential abundance analysis was performed using DESeq2 (Love et al., 2014). A model consisting of both genotype and treatment effect was used to test for differentially abundant ASVs. All statistical analyses on microbial community data were performed into the R environment, version 3.4.4 (R Core Team, 2018).

A Pearson correlation (bivariate) analysis was performed to determine the existence of a correlation among the average values of the Shannon and Inverse Simpson indexes and the chemical characteristics of the soil (pH, EC, CEC, TC, TOC, TN, and C/N). The two-tailed test (*p* < 0.05) was also completed by SPSS software.

## Results

**FIGURE 1:**
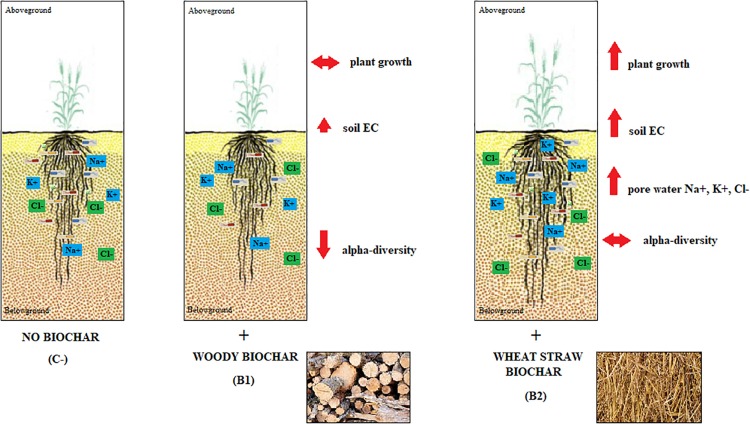
Schematic representation of the main effects of the biochar amendment observed in soil properties, plant growth, and rhizosphere bacterial richness. The more significant outcomes from the two biochars used, i.e., that from wood and that from wheat straw feedstock, are expressed in comparison also with the soil-only control (no biochar).

The characteristics of the two biochars used - wood chip feedstock for B1 and wheat straw for B2 – are shown in Table S1 in Supplementary Material S1, while the main features of the two durum wheat varieties analyzed – i.e., Duilio and Marco Aurelio – are listed in Supplementary Table S1 in Supplementary Material S2. The results of our study confirm that biochar amendment influences several of the features analyzed related to soil properties, plant growth, and rhizosphere bacterial microbiota (Figure 1), as reported in detail in the three sections below.

### Effect of Biochar Amendment on Soil Chemical Properties

**FIGURE 2:**
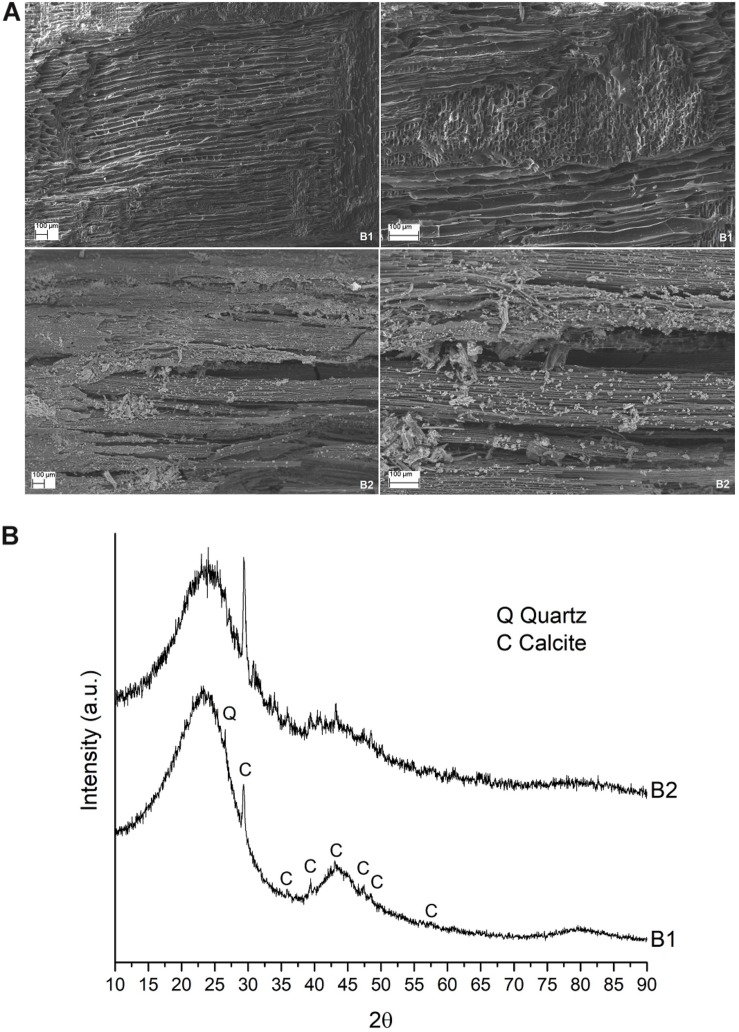
**(A)** Microstructure of the two biochars used. a SEM images of biochar B1 (up) and B2 (down), 100X and 250X on the left and right side, respectively. **(B)** X-ray diffraction pattern of the two biochars.

As expected from the physical and chemical features reported in Supplementary Table S1 in Supplementary Material S1, the two different biochars used exhibited different behavior. Indeed, when examined by scanning electron microscope, biochar from wood (i.e., B1) presented interconnected multi-directional channels with dimensions ranging from 5 to 50 microns, while biochar from wheat straw (i.e., B2) presented straight channels of 4–8 μm with pores of 3–4 μm (Figure 2A). On the other hand, they presented a very similar diffraction pattern, with broad peaks at about 3.85 Å, 2.09 Å, and 1.20 Å, which are generally associated with disordered carbons (Keiluweit et al., 2010). Some sharper peaks were also present in both biochar samples, mainly related to calcite (CaCO_3_) and quartz (SiO_2_) (Figure 2B).

The initial pH in water (5.17 at T_0_ in the soil alone) showed more than a unit of increase in all of the samples at the end of the experiment (T_f_) (Supplementary Table S1 in Supplementary Material S1). EC showed important variations between the soil at the beginning of the experiment (T_0_) and the soil and mixtures at the end (T_f_), apart from B1− samples at T_f_, where the EC value was similar to the initial control soil. In all other samples, EC showed an increase that was higher in B2- samples (Table 1). The CEC value increased in all samples at T_f_ with respect to the initial soil control, and a higher increase was related to the samples amended with activated wood chip biochar (average CEC value for B1+ samples was 6.02 ± 0.23 cmol/kg). The effect of biochar amendment on the carbon–nitrogen ratio (C/N) was remarkable, where the ratio was ≥22.6 for all treated variants and ≤8.0 for both control variants (against 14.4 for the initial soil). The TC and TOC concentrations (*mg*/*g*) at T_f_ were both significantly higher for all biochar treated variants in comparison to control. TN at T_f_ with biochar was always equal or higher than in controls. Total phosphorus (TP) was not reported in Table 1 because it was undetectable in all samples.

**TABLE 1:** Chemical characteristics and total metal concentration in soil at the beginning of the experiment (T_0_), and in soil and soil plus biochar mixtures at the end of the experiment (T_f_).

	**Sample**	**pH *(−)***		**EC *(uS m^–1^)***	**CEC *(cmol kg^–1^)***	**TC *(mg/g)***	**TOC *(mg/g)***	**TN (*mg/g*)**	**C/N *(−)***	**Total (available) element content *(mg kg^–1^)***									
									
		**H_2_O**	**KCl**							**Mg**	**Ca**	**K**	**Na**	**Al**	**Fe**	**Mn**	**Ba**	**Pb**	**Zn**
T_0_	Soil	5.17^∗∗∗^	4.08^∗∗∗^	73.8^∗∗∗^	3.53^∗∗∗^	2.87^∗∗∗^	2.31	0.20	14.4	2382 (111)	764^∗∗∗^ (283^a^)^∗∗∗^	982^∗∗∗^ (68)	84 (5)	13336^∗∗^ (11)^∗∗∗^	10778 (17)^∗∗∗^	610 (94)^∗∗∗^	104 (11)^∗∗∗^	53 (0)	48^∗∗∗^ (7.1)^∗∗∗^
T_f_	V1C	6.56	6.37	93.6	5.70	1.20^∗∗∗^	1.96	0.15	8.0^∗∗∗^	2669 (99)	2590 (58^a^)	1372 (22)^∗∗∗^	88 (18)	17331 (1.2)	10847 (4.2)	709 (11.1)	97 (4.8)	58 (0)	69 (0.5)
	V2C	6.53	6.35	103.1	5.72	1.16^∗∗∗^	1.94	0.16	7.3^∗∗∗^	2664 (99)	2522 (33^a^)	1633 (23)^∗∗∗^	117 (18)	18356 (1.1)	10148 (2.2)	686 (9.5)	99 (4.9)	54 (0)	69 (0.4)
	V1B1−	6.44	6.32	66.8^∗∗∗^	5.31	5.08	3.69	0.20	25.4	2512 (88)	2002^∗∗^ (35^a^)	1488 (67)	92 (16)	17522 (0.2)	9606 (0.1)	762 (8.3)	94 (6.5)	61 (0)	69 (<0.1)
	V1B1+	6.73	6.52	95.1	5.86	4.18	3.10	0.18	23.2	2541 (94)	2507 (16^a^)	1449 (51)	95 (21)	17175 (0.1)	9989 (0.2)	752 (4.9)^∗∗^	96 (5.1)	58 (0)	68 (<0.1)
	V1B2−	6.51	6.40	123.2	5.60	4.30	2.60	0.19	22.6	2653 (84)	2073^∗∗^ (122^a^)^∗∗∗^	2044^∗∗∗^ (292)^∗∗∗^	107 (24)	19030^∗∗^ (0.2)	10284 (0.2)	779 (8.8)	100 (6.5)	62 (0)	73 (<0.1)
	V2B1−	6.40	6.26	74.6^∗∗∗^	4.84^∗∗^	4.94	3.97	0.18	27.4	2505 (88)	2044^∗∗^ (14^a^)	1431 (66)	100 (19)	17878 (0.2)	9638 (0.1)	806 (9.5)	95 (6.7)	61 (0)	72 (0.1)
	V2B1+	6.73	6.55	91.5	6.18^∗∗^	4.25	2.96	0.15	28.3	2537 (93)	2505 (22^a^)	1538 (49)^∗∗∗^	104 (21)	17533 (<0.1)	9661 (0.1)	763 (4.9)^∗∗^	99 (4.7)	60 (0)	73 (0.1)
	V2B2-	6.56	6.34	129.6	4.91	3.77	3.52	0.17	26.9	2598 (84)	2046^∗∗^ (61^a^)^∗∗^	1887^∗^ (298)^∗∗∗^	99 (24)	18132 (0.1)	10240 (0.1)	768 (8.1)	96 (6.5)	61 (0)	70 (0.1)

*Data shown are means ± SD (*n* = 2). Significant differences (*p*-value) ^∗∗∗^ < 0.001, ^∗∗^ < 0.01, ^∗^ < 0.05. Values in brackets represent available form of the elements using 0.01 M CaCl_2_; ^*a*^values containing Ca coming from 0.01 M CaCl_2_; EC is electrical conductivity; CEC is cation exchange capacity; TC is total (organic plus inorganic) carbon; TOC is total organic carbon; TN is total nitrogen; C/N is the ratio between carbon content and nitrogen content.*

The total content of several elements in the soils was measured by the *aqua regia* extraction method (Table 1). In addition, the bioavailable content of these elements in the soils was determined by the CaCl_2_ extraction method (Table 1, values in brackets). In summary, all of the total cation (Mg, Ca, K, and Na) contents increased slightly during the experiment, but at the same time, the available form of the cations decreased in a similar fashion, except Na. It is interesting that, at T_f_, in the presence of B2−, K^+^ ions in soil and particularly those available to the plant showed a substantial increase (close to 300 mg/Kg of usable potassium in B2-samples, ranging from 50 to 70 mg/Kg in B1 treated samples, and just over 20 mg/Kg in control samples without biochar). Total aluminum (Al) increased in all samples at T_f_, while the available Al decreased, and the presence of biochar enhanced this Al decrease. Moreover, the presence of biochar resulted in a decrease of bioavailable Fe, Mn, and Ba. Concerning heavy metals, no lead usable by the plant was detected in any sample, either at T_0_ or T_f_, and a small increase in total Pb was seen due to the biochar amendment. Total zinc (Zn) showed an increase during the experiment, independently of the presence of biochar, whereas the Zn available to plants decreased substantially at the end of the experiment, and biochar-treated samples showed lower amounts.

Rhizones were used to collect pore water from soil pot samples at the end of the experiment. The values for pH and EC obtained from the pore water samples were comparable with those from the soil samples. Furthermore, the concentrations of several nutrient ions in the pore water samples were also determined (Table 2). Major variations were found in B2- treated samples showing significantly higher amounts of Na^+^, K^+^, and Cl^–^ ions. B1+ treated samples showed a slight increase in Mg^2+^ ions. Despite the improvement of B1+ by Hoagland solution, there were no significant differences between B1+ and the two untreated biochars through all of the nutrients analyzed in the final pore water. Moreover, the contents of dissolved phosphate anions in pore water were very low (Table 2).

**TABLE 2:** Concentration of the main ions in pore water samples collected by rhizones at the end of the experiment.

	**pH *(–)***	**EC *(mS cm^–1^)***	**Dissolved ions in pore water *(mg L^–1^)***								
			
			**Na^+^**	**Mg^2+^**	**K^+^**	**Ca^2+^**	**Zn^2+^**	**Cl^–^**	**NO_2_^–^**	**NO_3_^–^**	**SO_4_^2–^**
V1C	6.39 ± 0.01^a^	0.17 ± 0.03^bc^	5.85 ± 0.89a	3.89 ± 1.14	1.31 ± 0.40^a^	68.7 ± 8.67	0.88 ± 0.66	9.68 ± 7.86^ab^	3.06 ± 1.16	79.1 ± 43.2	61.2 ± 35.6
V2C	6.57 ± 0.07^a^	0.17 ± 0.02^bc^	4.48 ± 0.30^a^	4.44 ± 0.34	3.23 ± 2.94^a^	67.5 ± 1.78	0.63 ± 0.08	5.46 ± 2.72^a^	1.69 ± 0.70	67.3 ± 5.3	73.6 ± 3.3
V1B1−	6.47 ± 0.01^a^	0.11 ± 0.04^c^	5.85 ± 1.19^a^	4.11 ± 1.55	5.10 ± 0.90^a^	60.2 ± 20.29	1.04 ± 0.59	6.86 ± 8.13^ab^	2.69 ± 1.03	112 ± 75	118 ± 100
V1B1+	6.58 ± 0.07^a^	0.35 ± 0.11^ab^	9.07 ± 0.75^a^	7.14 ± 2.07	4.32 ± 0.78^a^	103 ± 28.69	1.36 ± 0.99	9.76 ± 0.95^ab^	4.87 ± 1.65	125 ± 47	79.8 ± 14.1
V1B2−	6.45 ± 0.07^a^	0.36 ± 0.04^a^	12.9 ± 1.3^b^	5.36 ± 0.78	54.3 ± 9.98^b^	65.8 ± 4.18	0.78 ± 0.22	37.8 ± 11.9^abc^	4.47 ± 2.41	80.0 ± 31.9	76.8 ± 16.3
V2B1−	6.63 ± 0.09^a^	0.16 ± 0.03^bc^	6.66 ± 0.56^a^	3.39 ± 0.19	14.2 ± 6.24^a^	55.7 ± 10.34	0.78 ± 0.17	9.44 ± 6.82^ab^	3.00 ± 1.26	73.4 ± 20.0	42.2 ± 15.6
V2B1+	6.96 ± 0.15^b^	0.24 ± 0.08^abc^	8.73 ± 1.36^a^	5.59 ± 1.30	6.02 ± 1.71^a^	75.6 ± 12.31	0.46 ± 0.29	13.5 ± 7.04^ab^	5.11 ± 3.30	79.5 ± 14.2	93.9 ± 19.8
V2B2−	6.33 ± 0.10^a^	0.43 ± 0.06^ab^	12.9 ± 3.3^b^	4.18 ± 1.62	53.9 ± 18.39^b^	77.1 ± 14.38	1.15 ± 0.76	38.0 ± 15.5^bc^	4.68 ± 2.11	133 ± 86	85.0 ± 42.8
*p*	<0.001	0.005	< 0.001	0.188	<0.001	0.165	0.796	0.006	0.582	0.804	0.807

*Data shown are means ± SD (*n* = 3). Different letters in a single column indicate a significant difference among the samples analyzed (*p* < 0.05).*

### Responses of Plant Growth and Biomass Production to the Presence of Biochar in Soil

At the final time of the experiment (T_f_), the phenological stage for all plants according to the Feekes scale of wheat development was assessed at 10, corresponding to boot exposure at the end of stem extension and before heading. On average, each plant showed one tiller and 7–8 leaves.

**FIGURE 3:**
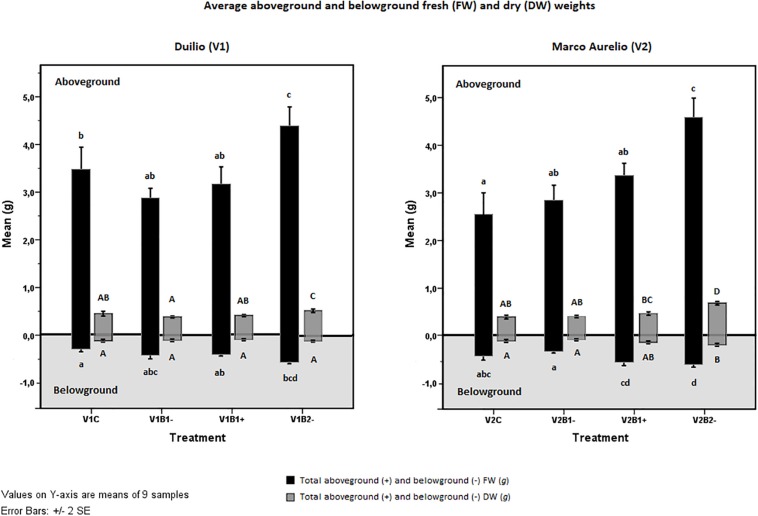
Effect of genotype and treatment on fresh weight (FW, black bars) and dry weight (DW, dark gray bars) in the aboveground plant (upper part on white background) and in the belowground root system (lower part on the light gray background). Duilio data is on the **left**; Marco Aurelio data is on the **right**. Letters close to the error bars indicate the homogeneous subsets resulting from Tukey HSD *post hoc* test (*p* < 0.05). Lower case letters refer to FWs and upper case to DWs.

The possible effect of the treatments applied (C, B1−, B1+, B2−) on plant growth and biomass was evaluated through the fresh (FW) and dry (DW) weights, specifically for the two durum wheat varieties under study, and for the aerial part of the plant and the rooting system. Concerning the plant total aboveground fresh weight (tAGFW), there was a significant difference (*p* < 0.05) between Duilio (3.468 ± 0.714 g) and Marco Aurelio (2.542 ± 0.691 g) control plants (Figure 3, V1C and V2C, black bars in the positive panel), as expected from previous experiments (unpublished data). This difference was not evident in the total aboveground dry weight (tAGDW) of the control plants (Figure 3, V1C and V2C, dark gray bars in the positive panel). Non-activated biochar 2 (B2−) exhibited a significant positive effect on both varieties (*p* < 0.001), with a tAGFW increase of up to 4.408 ± 0.620 g and 4.582 ± 0.612 g, respectively, in Duilio and Marco Aurelio (Figure 3, V1B2− and V2B2−, black bars in the positive panel). On the other hand, the effects of biochar 1, either activated or not, on the aerial plant FW were not significant (again Figure 3). Total aboveground dry weight (tAGDW) showed a similar trend, increasing in both varieties with B2−. The mean difference between control and B2− in Duilio was 0.095 g (*p* < 0.01) and in Marco Aurelio was 0.289 g (*p* ≥ 0.000), with a Std. Error of 0.025 (Figure 3). In conclusion, B1 (activated or not) and B2 (not activated) behaved differently with respect to the aerial plant weight: the addition of B1 to soil did not result in any considerable change in the fresh or the dry weight of the plant aboveground fraction; in contrast, B2-addition to soil in its untreated form was found to be related to a statistically significant increase in these parameters.

Looking at the results related to the root system fresh weight (tBGFW), a positive effect of B2− on both Duilio and Marco Aurelio was again evident: here, the mean difference between control and B2− in Duilio was 0.246 g (*p* ≥ 0.000), and that in Marco Aurelio was 0.172 g (*p* < 0.01), with a Std. Error 0.045. Moreover, exclusively for Marco Aurelio, the effect of B1− differed significantly from that of B1+ (*p* ≥ 0.000), with a mean difference between V2B1− and V2B1+ equal to 0.232 g, and a Std. Error of 0.045 (Figure 3, black bars in the negative panel). Lastly, the total belowground dry weight (tBGDW) was significantly increased only in Marco Aurelio plants grown in B2− pots. More precisely, the average DW value of V2B2− in all replicated samples was significantly higher than in other treatments (*p* < 0.01), except for V2B1+, where this difference is not significant (*p* = 0.113) (Figure 3, dark gray bars in the negative panel). See Supplementary Material S7 for the results of the statistical analysis carried out with the SPSS tool.

**FIGURE 4:**
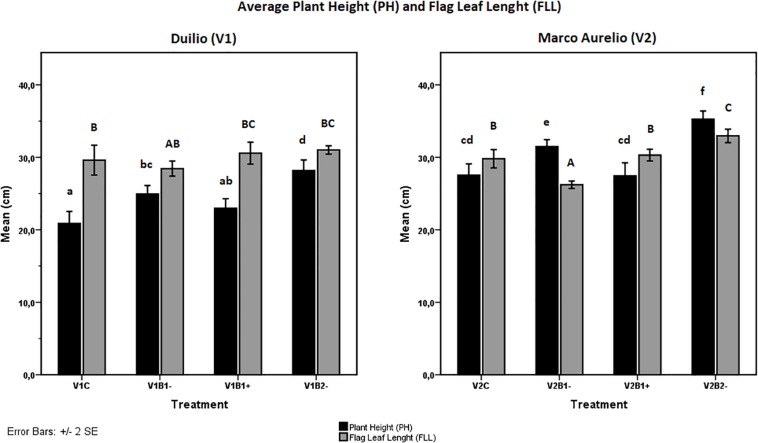
Effect of genotype and treatment on plant height (PH, up) and flag leaf length (FLL, down) in Duilio (V1, on the **right**) and Marco Aurelio (V2, on the **left**). Letters close to the error bars indicate the homogeneous subsets resulting from Tukey HSD *post hoc* test (*p* < 0.05). Lower case letters refer to PH and upper case to FLL.

As additional traits for the evaluation of plant growth performance, PH, FLL, and maximum width (mFLW) were also measured at the end of the experiment (T_f_). Concerning PH, even though exhibiting different heights in the control situation, lower in Duilio and higher in Marco Aurelio, both cultivars showed a similar positive influence from biochar treatment; indeed, the height increases resulting from B1− in a sharper fashion (*p* < 0.003 in V1 and *p* < 0.004 in V2) and B2− in a stronger fashion (*p* < 0.000 in both V1 and V2) were highly significant (Figure 4, black bars). The FLL trait was not significantly influenced by biochar in Duilio (*p* > 0.05), but was significantly altered by B1− (in a negative way; *p* < 0.002) and B2− (in a positive way; *p* < 0.009) in Marco Aurelio (Figure 4, gray bars; Supplementary Material S8). Lastly, the mFLW values were not normally distributed, so they were not considered for further statistical analysis; notwithstanding, in Marco Aurelio plants treated with B2−, flag leaf presented an increased width (data not shown).

The genotype influence on the plant response to biochar treatment (B1−, B1+, B2−) with respect to the control without biochar was also assessed. Table 3 lists the plant response (“+” indicates a significant augmentation, “−” indicates no significant effect) in relation to the plant growth traits measured at the end of the experiment under the different treatments. The information reported in the table proceeds directly from the homogeneous subsets defined by Tukey HSD test (see Figure 3 and related Supplementary Material S7, and Figure 4 and related Supplementary Material S8). On the bases of the considered traits, the genotype-dependence is particularly evident in the case of B2−, where both V1 and V2 showed a general trend of plant growth increase, but their responses differentiate in relation to a few traits. In this case, tBGDW, FLL, and mFLW were kept almost constant in V1 while they were positively affected in V2 (Table 3).

**TABLE 3:** Genotype influence on the plant response to biochar treatment.

	**B1–**		**BB1+**		**B1−V**	
			
**Measured plant growth traits**	**V1**	**V2**	**V1**	**V2**	**V1**	**V2**
tAGFW	−	−	−	−	+	+
tAGDW	−	−	−	−	+	+
tBGFW	−	−	−	+	+	+
tBGDW	−	−	−	−	−	+
PH	+	+	−	−	+	+
FLL	−	+	−	−	−	+
mFLW	N.A.	N.A.	N.A.	N.A.	−^∗^	+^∗^

*“+” indicates a significant positive augmentation with respect to control at T_*f*_, and “−” indicates no significant effect with respect to control; N.A. is not available: ANOVA statistics were applied to mFLW because the measured were not normally distributed; ^∗^ results of ANOVA statistics for data that were not normally distributed.*

### Effect of Biochar Soil Amendment on Rhizosphere Bacterial Community Structure and Diversity

**FIGURE 5:**
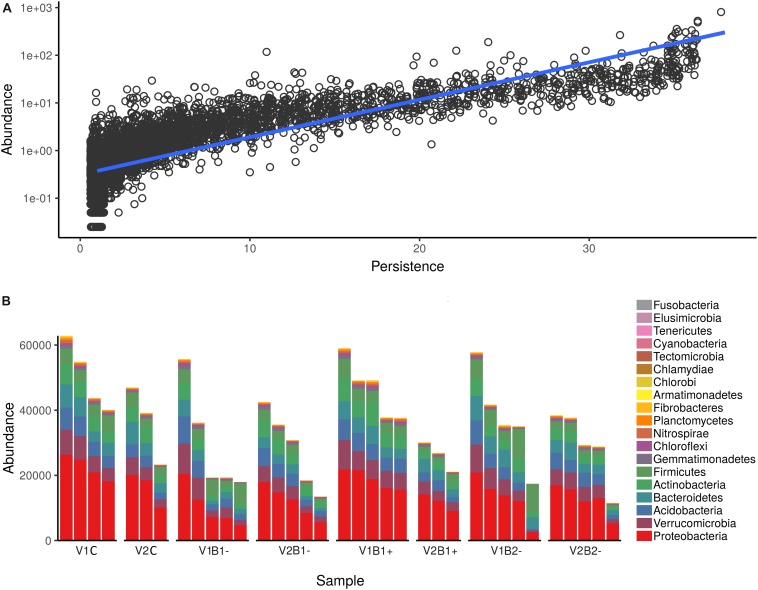
Abundance of the 16S rRNA gene ASVs in the rhizosphere samples. **(A)** Persistence (on the *x*-axis) and abundance (on the *y*-axis) of ASVs detected in all samples. Persistence is expressed as the number of samples in which a given OTU (Operational Taxonomic Unit) was detected, whereas abundance is expressed as log-normalized number of reads assigned to a given ASV (linear regression is shown as a blue line; *R*^2^ = 0.66, *F*(1,6126) = 11,947.91, *p* < 0.001). **(B)** Absolute abundance of ASVs. All ASVs were collapsed at the Phylum level and then plotted. Each bar reports the number of reads assigned for each sample colored according to the Phylum attribution. Plots are split according to treatment (top) and plant genotype (bottom).

To study the composition of bacterial communities in the rhizosphere samples, we used 16S rRNA amplicon sequencing. For a bacterial community, the number of specimens in which its members are detected (persistence) should be correlated to the abundance of those members, which is usually expressed as the normalized number of reads assigned to a given ASV (Shade and Handelsman, 2012; Bacci et al., 2015, 2018). In agreement with this definition, the abundance of all ASVs in different sampling sites was significantly correlated with the number of sites inhabited by those ASVs (linear regression on log-transformed abundance; *R*^2^ = 0.66, *F*(1, 6126) = 11,947.91, *p* < 0.001) (Supplementary Table S5 in Supplementary Material S9 and Figure 5A). The ASVs of bacteria belonging to the phyla Proteobacteria, Verrucomicrobia, Acidobacteria, Bacterioides, Actinobacteria, and Firmicutes were the most commonly present, as reported in Figure 5B.

**FIGURE 6:**
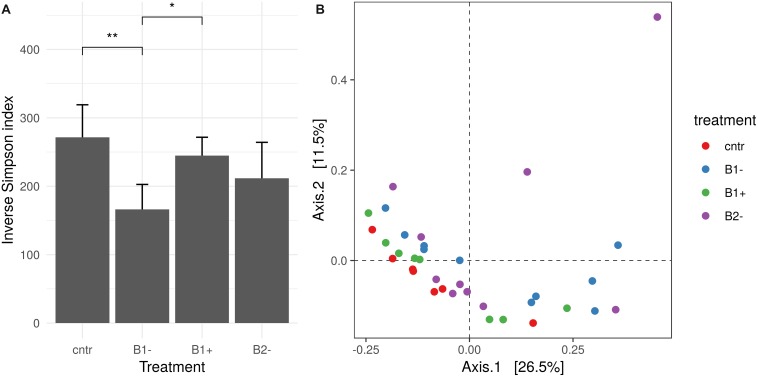
Rhizosphere bacterial diversity analysis. **(A)** Inverse Simpson index distribution for different treatments. Treatments that showed a significant difference are marked with an asterisk (Wilcoxon test, adjusted *p*-value using a “false discovery rate” lower than 0.05). **(B)** Principal coordinate analysis (PCoA) based on the Bray–Curtis index. Different colors report different treatments.

Alpha diversity was calculated to gain further insights into the complexity of the rhizosphere bacterial communities. It showed significant differences according to treatment but not according to genotype [two-way analysis of variance ANOVA; *F*(3,27) = 3.22, *MSE* = 5,512.21, *p* = 0.039 and *F*(1,27) = 0.38, *MSE* = 5,512.21, *p* = 0.542; Supplementary Figure S5 in Supplementary Material S9). No significant effect was detected for the treatment-genotype interaction [*F*(3,27) = 0.65, *MSE* = 5,512.21, *p* = 0.591; Supplementary Table S6 in Supplementary Material S9]. The inverse Simpson index was also used: alpha-diversity among treatments, independently on the genotypes, appears to be significantly lower under B1− treatment and to be kept almost stable under B1+ and B2− treatments, these last not varying with respect to the control (Figure 6A). In contrast, beta diversity showed a significant effect of treatment as well as genotype, with no significant interaction effect (permutational multivariate analysis of variance using distance matrices with 1000 permutations on the Bray–Curtis index; *p* = 0.001, *p* = 0.0009, *p* = 0.054, respectively). Despite the significant effect reported, the *R*^2^ value is quite low, highlighting that the percentage of variance influenced by the treatment and genotype factors is, in turn, low (*R*^2^ = 0.19 and 0.06, respectively) (Supplementary Table S7 in Supplementary Material S9). This effect is clear looking at the principal component analysis (Figure 6B), where samples are difficult to group based on the treatment used.

**FIGURE 7:**
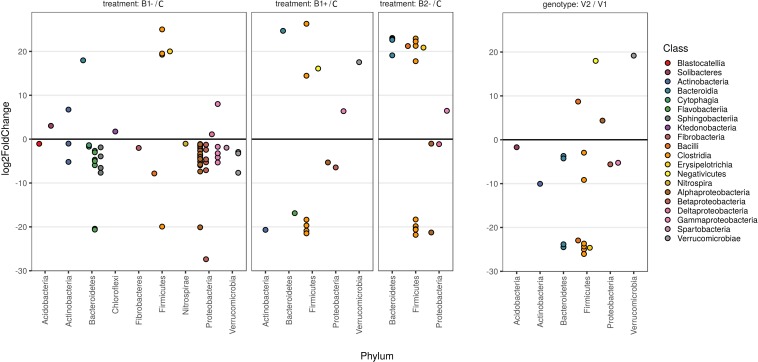
Fold changes of differentially abundant ASVs. The first three panels represent the comparison of a particular treatment with the control, while the panel on the right is a comparison between the two genotypes used (V2 *vs*. V1), as reported at the top of the plots. Each dot represents an amplicon sequence variant (ASV). Fold changes were calculated according to the contrast reported on the top of each panel and transformed into log2. Log2 fold change values are reported in the vertical axis so that positive values represent ASVs that are more abundant in the treatment group and vice versa. Only contrasts with a *p*-value lower than 0.05 and with a |log2-fold-change| higher than 1 are reported in the plot (see Supplementary Material S8). The phylum attribution for each ASV is reported on the horizontal axis, whereas colors refer to the Class. Colors are ordered based on the Phylum attribution to help the plot to be understood correctly.

Differential abundance analysis showed variation in the rhizosphere microbiome due to the treatment used (B1−, B1+, and B2−) with respect to the control as well as between plant genotypes (V2 *vs*. V1) (Figure 7). In particular, the biodiversity loss in B1− samples was confirmed by the reduction of numerous ASVs, especially those related to the classes of Alpha-, Beta-, and Gammaproteobacteria (Proteobacteria phylum), the classes of Cytophagia, Sphingobacteriia, and Flavobacteriia (Bacterioides phylum), and the class of Verrucomicrobiae (Verrucomicrobia phylum) (Figure 7, panel B1-/C). The details on the ASVs resulting from the differential analysis are provided in Supplementary Material S10.

**FIGURE 8:**
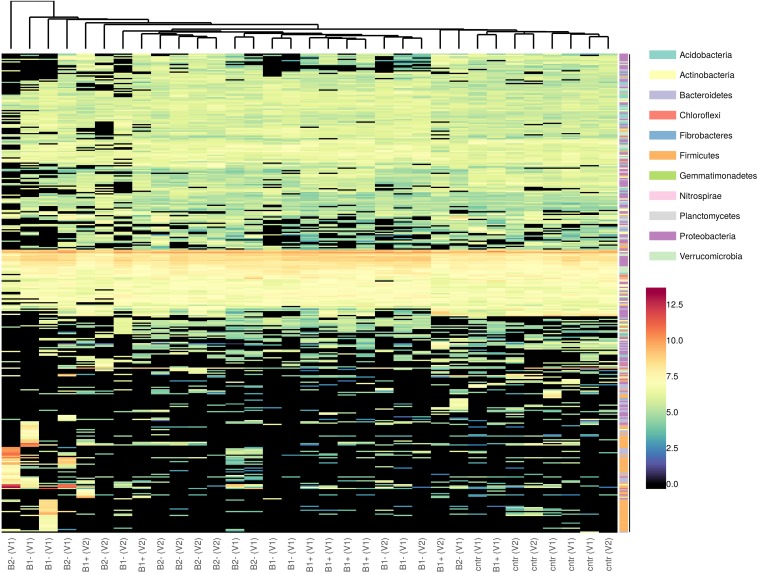
Heatmap of the taxonomic distribution of consistent ASVs. Taxa (the rows on the heatmap) were marked with different colors according to Phylum attributions. Colors on the far left of the plot refer to the Phylum attribution of each ASV. Counts were variance-stabilized and log2-transformed before plotting using DESeq2 normalization.

The taxonomic distribution of the consistent ASVs is shown in Figure 8. Even though, by looking at the reported hierarchical clustering, samples could neither be grouped based on treatment nor on genotype, a shared set of ASVs can be detected.

The correlation analysis between the Shannon and the inverse Simpson index, chosen as biological indicators of the diversity of microbial communities in the soil rhizosphere (Kim et al., 2017), and the chemical properties of the sampled soils highlighted a few significant strong negative associations (*p* < 0.05). In particular, the rhizosphere α-diversity was inversely related to the TC content (Pearson *r* equals −0.735 with Shannon index and −0.801 with Inverse Simpson index), and also to the TN content (in this case, only with the Inverse Simpson index, Pearson *r* equals −0.755), as shown in Supplementary Table S9 in Supplementary Material S11.

## Discussion

According to the research literature and also to experience, biochar amendment of soil may influence soil properties, plant physiology, and other environmental traits, including microbial composition at the levels of plant, soil, and rhizosphere. Here, we focused our attention on three main features – soil chemical composition, plant growth in the early growth stage, and rhizosphere bacterial microbiome – and hypothesized that the likely effects of biochar may be different depending on the biochar and the plant genotype. It is well established that the physicochemical properties of biochar and, for instance, its effects on the environment are strictly linked to the feedstock material used for its manufacture (Lei and Zhang, 2013; Shen et al., 2017b). Another very important factor is the temperature used during pyrolysis, even though Zhang et al. (2017) found that not all biochar properties change consistently with increasing temperature. Thus, to attain further information and determine to what extent the original vegetal source of the biochar could contribute to determining its overall effect, we used two biochars produced from two different feedstocks (wood and wheat straw) but at the same high pyrolysis temperature (around 700°C). Furthermore, the effects of biochar strongly depend on plant species and also on cultivars within a species, and even though their determination is fundamental for the most advantageous implementation of biochar in agriculture, they are still poorly known (Mollinedo et al., 2016; French and Iyer-Pascuzzi, 2018). Our results showed that the soil, plant, and rhizosphere microbiome are influenced by biochar addition, underlining the complexity of the effects of biochar and the resulting soil and plant reactions.

### Biochar Amendment Increases Soil Carbon Content, pH, and CEC

Our results confirm that biochar amendment has a broad influence on the physical and chemical properties of the soil. As expected, we observed a remarkable effect of biochar on TC, TOC, and C/N, whose values were markedly higher in the biochar-treated samples than in the controls. In particular, the increased C/N ratio in biochar-treated variants is related to higher microbial activity (Wan et al., 2015), and, as was obvious from our results, application of biochar set up the conditions for an ideal microbial diet (C/N ≈ 24:1; USDA, 2011) during the experiment, unlike in the control (C/N ≤ 8.0; see Table 1). On the other hand, we did not expect an increase in pH and CEC with respect to the sample soil in V1C and V2C, which are the control samples made of soil only, at the final time of the experiment (T_f_). In fact, we foresaw such an increase exclusively for the samples treated with biochar. The difference in pH and CEC may be due to many factors that alter the soil under greenhouse conditions, including plant growth and local and root microorganisms that developed in the soil pot. We used demi-water for plant irrigation, and here we can speculate that root exudates caused a buffering effect (Trakal et al., 2017). It has been proved that root exudates affect the pH in the rhizosphere: Smiley (1974) found an increase in soil pH in the presence of crops; Romkens et al. (1999) found that the increase in pH was related to the excretion of OH^–^ by the roots during N and Ca uptake.

### Biochar From Wheat Straw Increases EC and Nutrient Availability in Soil and Monovalent Element Ions in Pore Water

In particular, B2 coming from wheat straw feedstock is a biochar with a higher nutrient content with respect to B1 from wood, as shown in Supplementary Table S1 (e.g., for total content of nitrogen and/or phosphorus). This is also the reason why we applied B2 directly to the soil without a previous “activation” in a nutrient solution (B2− treatment), as was additionally performed with B1 (both B1− and B1+ treatments were assayed). Looking at the total metal contents, and particularly at the bioavailable element contents (Table 1), which better represent the nutrient substrates that can directly benefit and sustain plant growth, B2− amendment showed a stronger impact on the pots in both the Duilio and Marco Aurelio varieties, leading to higher levels of available nutrients (mainly very high contents of usable K and Na with respect to all other soil pots). Furthermore, we used by rhizones to analyze the nutrient concentration in the soil solution occupying the interstitial spaces (Table 2), and B2− treatment resulted in a substantial increase in the concentration of sodium, potassium, and chloride ions dissolved in water, which explains the more nutrient-rich composition of the wheat straw biochar against the woody one. The biochemical functions of these elements used to be related to their osmotic potentials, controlling membrane permeability and regulating the cell osmotic tone and ionic balance. Surprisingly, there were no significant changes in nitrate (NO_3_^–^) concentrations among all variants [even between (non)activated biochars, B1+ *vs*. B1−], which could be explained by individual N uptake into durum wheat through all variants (represented by very high SD).

It is worthy of note that our experiment gives an additional proof of the capability of biochar to decrease Zn availability for the plants (Table 1), thus supporting its application for the remediation of soils from zinc and other heavy metal pollutants (Zhang et al., 2013; Puga et al., 2015). The Zn concentration in pore water, nevertheless, does not fit this conclusion. This can probably be explained by significant dissolution of Zn by root exudates and consequent uptake into the biomass through all variants (during the whole period of the experiment). Indeed, zinc is needed by plants in small amounts, and it is crucial to plant development, playing an important role in a wide range of biochemical processes. Here, only a limited amount of the available Zn was then extracted by 0.01 M CaCl_2_ from the final soils in which that Zn was: (*i*) already consumed by plants, and/or, most probably, (*ii*) Zn ions were most strongly bound to the biochar surface (Kiekens, 1995; Trakal et al., 2017).

### Wheat Straw Biochar Enhances Durum Wheat Biomass

As broadly reported in literature, biochar amendment may also play an important role in plant growth, and the resulting effect is often augmentative [Baronti et al. (2010) and Vaccari et al. (2011) specifically for durum wheat; Solaiman et al. (2010) and Meng et al. (2019) for bread wheat; Kammann et al. (2015) for quinoa; and several others], but it may also be diminutive [as for example in Aguilar-Chávez et al. (2012) for bread wheat and Kammann et al. (2015) for quinoa] or even lead to no significant variation [as in Tammeorg et al. (2014) and Kelly et al. (2015) for bread wheat; and others].

In particular, in our experiment, biochar from wheat straw in its non-activated status (B2−) was related to a significant increase in plant fresh and dry weights, in both tested cultivars, with the only exception being Duilio root DW, which stayed almost constant among all treatments. This exception is not so unique, and indeed it has also been reported that aboveground productivity is one of the positive significant effects of biochar, while belowground productivity remains unaffected (Biederman and Harpole, 2013). To date, the effects of biochar application on root traits are still controversial, and results are highly variable (Xiang et al., 2017).

As reported in the results, B2− treatment markedly impacted soil nutrient status, and the major increase in soil potassium content was significant. Since potassium represents an element of huge importance to ensure healthy and well-sustained plant growth and participates in several metabolic pathways (Prajapati and Modi, 2012), one could hypothesize that the higher plant growth in the presence of wheat straw biochar was also attained thanks to the potassium increase directly determined by the biochar.

### Long-Term Effects of Biochar on Plant Growth Need to Be Investigated

It is worthy of note that biochar from wood chips (B1) had no relevant effects on plant biomass (neither above- or belowground), independently of the way the biochar was supplied, i.e., pure, untreated, and coming directly from fresh production (B1−) or previously activated in a nutrient-rich solution (B1+). Concerning B1−, under greenhouse conditions and in the frame of the 6-week duration of our experiment, which may be considered a short time, one could expect that all of the biochar active sites, initially free at T_0_, would have immediately bound to several nutrients; moreover, the use of a soil with a low SOM could even have led to plant starvation. In contrast, B1+ could be somewhat compared to co-composted biochar, and one could hypothesize this to determine a stronger effect on plant growth. In fact, it has been ascertained that co-composting improves the plant growth-promoting effects of biochar (Kammann et al., 2015). In our settings, we did not achieve the expected results, and a significant positive effect from either the B1− or B1+ treatments was lacking. In any case, the current experiment draws attention to the response of the durum wheat plant to biochar amendment during its first stages of development and growth. Thus, we designed pot experiments in a greenhouse under controlled conditions because this is easier to perform in order to infer preliminary results and avoids the complications that may arise due to the overlap of several environmental effects as happens in the field. It is possible that, in the field and with a longer experimental duration, with the aim of measuring the effects of durum wheat yield not only in terms of shoot and root biomass but particularly in terms of grain yield, the plants would have shown different behavior because of other factors not considered here.

### Biochar From Straw-Based Feedstock Is More Suitable Then Woody Biochar for Improving Crop Yield

The significant effect of B2 on plant growth and the lack of effect of B1 is in perfect accordance with the results of the comparative study of the properties of biochar from wood material and crop residues performed by Wang et al. (2013), who showed that, despite high variability, biochars from crop straw may be more effective and desirable for improving soil fertility. In crop-residue-based biochars, including wheat straw-based biochar, they found a higher ash%, CEC, total contents of N, P, Ca, and Mg, pH, and wt% of C, Na, and K than in wood-based biochars. Many of these properties are comparable with the data reported in Supplementary Table S1. Moreover, they found that the BET surface area of straw-based biochars with a 700°C pyrolysis temperature, which is the same temperature used for the production of the two biochars analyzed in our experiment, may be wider than for other biochars (Wang et al., 2013). In this context, the scanning electron microscopy visualization of the biochar samples used (Figure 2) let us infer that the structure of B2, including its porous arrangement, distribution, and size, creates a larger surface area that improves the connections between plant and soil, allowing the plants to enhance their growth, at least in terms of fresh dry weight (with the only exception being Duilio tBGDW; Figure 3) but also in terms of average PH (Figure 4).

### Durum Wheat Growth Response to Biochar Treatment Is Genotype-Dependent

In regard to a genotype dependence of plant response to the biochar treatments, there is proof that, for each treatment, both genotypes exhibited a general trend of response with some cultivar-specific exceptions. It is relevant that Marco Aurelio is more positively responsive to B2−. In fact, Marco Aurelio increases the magnitude of all measured traits (tAGFW, tAGDW, tBGFW, tBGDW, PH, FLL, and mFLW), while Duilio shows a significant increase due to B2− in most of these traits but not in all (tBGDW, FLL, and mFLW are not significantly influenced). As has emerged from other studies [Chen et al. (2016) and French and Iyer-Pascuzzi (2018) as examples], this result confirms that, where the aim is simultaneously improving soil fertility and increasing crop yield, for every target crop, the choice of the best cultivar genotype that better responds and adapts to a particular biochar is critical.

### Biochar Treatment but Not Durum Wheat Genotype Has a Significant Influence on Rhizosphere α-Diversity

To assess the possible changes caused by biochar incorporation and/or the durum genotype to the soil microbiome, we specifically focused on the rhizosphere, which is the interface between plant roots and soil, since the microorganisms colonizing the rhizosphere may contribute to plant growth and health (Richter-Heitmann et al., 2016); moreover, different studies have already established that the rhizosphere corresponds to the plant-soil compartment harboring a higher richness (Qian et al., 2019).

Rhizosphere soil samples, like the soils, are very complex matrixes, and high-throughput 16S rRNA amplicon sequencing produced data that was difficult to analyze statistically, due in particular to the “background noise,” which makes it hard to clearly distinguish what is really influenced by the treatment and by the genotype. Notwithstanding, bacterial species richness and their abundance levels, as may be inferred by the alpha diversity, showed influences due to the biochar treatment, while no significant change was encountered when assessing the effect due to the durum wheat variety (in contrast with our assessed genotype effects on plant growth). In the literature for bread wheat, it is reported that host genotype played a minor but significant role in the bacterial diversification of the rhizosphere (Mahoney et al., 2017); and, indeed, genotype is generally thought to have a minor role in shaping microbiota composition (Bulgarelli et al., 2012; Kwak et al., 2018). The bacterial phyla encountered in the rhizosphere samples analyzed and their abundances are consistent with those most commonly present in similar samples, as reported in different published research articles (Mahoney et al., 2017; Wang et al., 2019) and include Proteobacteria, Verrucomicrobia, Acidobacteria, Firmicutes, Bacterioidetes, and Actinobacteria. In agreement with Mahoney et al. (2017) and other previous studies on the wheat rhizosphere, we also found the Sphingobacteriia class as a dominant taxon.

### Low-Nutrient Biochar Negatively Impacts Rhizosphere Microbiota Species Richness

In the rhizosphere of the untreated woody biochar (B1−) samples, a lower inverse Simpson index indicated a lower species richness and evenness, while in the other treatments (B1+ and B2−), the value of this index did not vary significantly (Figure 6). This outcome is worthy of note because it means that a nutrient rich biochar, such as B1+ and B2−, under the experimental conditions set up, does not negatively impact the rhizosphere bacterial species richness and keeps it stable. This result highlights the key role played by a biochar and a soil in shaping the microbiota, altering the number of species present and the relative abundance of each species, or keeping them stable and balanced. The outcome that a nutrient-rich source may favor the maintenance of a balanced bacterial microbiota status has also been shown in other studies; for example, Qiao et al. (2017) found, in cotton, that bacterial α-diversity in the rhizosphere of nutrient-rich soil was lower than in a soil from a continuous cropping field, while β-diversity was greater. In a different way, for bread wheat it has just been reported that wheat straw biochar increases the biodiversity of the soil microbiome and also the mycobiome of the seedlings’ rhizosphere under herbicide stress, with the percentage of biochar application to the soil influencing the soil microbial community structure (Meng et al., 2019).

### Both Biochar Treatment and Durum Wheat Genotype Influence the Overall Bacterial Composition of the Rhizosphere

In another way, our study highlighted a compositional difference of the bacterial microbiota expressed in terms of ASVs, as may be presumed by the beta-diversity, not only among the treatments but also between the two genotypes analyzed. The comparison among treatments and genotypes highlighted interesting variations related to the abundances of the different ASVs. In our experiment, we could not find any significant change attributable to a specific taxon (at least at the levels from Phylum to Genus), and the observed variation was due to several sequence variants that may represent single nucleotide variations, i.e., a particular strain of a species (Figure 8 and Supplementary Material S10). This is also what we expected based on the fact that our pot samples were very similar with each other (same soil, same plant species, same irrigation level, same greenhouse conditions, etc.), with the only exception being the applied biochar, when comparing with the control to detect the effect of the treatment, or of the plant genotype, when comparing the two durum wheat varieties with each other. Moreover, concerning the different biochar applied, the bacterial input coming from both B1 and B2, both dried, was assumed to be very limited. In this case, one does not expect a big variation at the level of Phyla or Class but may hypothesize that there will be much variation in species and/or within species.

From the higher C/N ratio detected in the bulk soil of the treated samples at the end of the experiment, we could expect an increased microbial activity. Our analysis of the rhizosphere does not allow us to confirm this occurrence, not only because we analyzed the rhizosphere compartment only but also because the degree of diversity could not necessarily be linked to the level of microbial activity, which should be measured spectrophotometrically through enzymatic activity or through soil respiration.

### Influence of Soil Chemical Properties on the Diversity of Rhizosphere Bacterial Communities

In our study, Pearson’s correlation analysis was adopted to estimate the associations between soil chemical characteristics and rhizosphere bacterial diversity indexes, revealing a strong negative correlation of microbiological indexes with soil TC and TN. Soil microorganisms, especially bacteria, which represent the most abundant group, play central roles in ecosystems. Even though several studies report that soil pH, TC, TOC, and TN are among the main abiotic factors structuring bacterial communities, they are not necessarily associated with the soil microbiota (Deng et al., 2018), particularly in rhizospheric soil; moreover, it is also well known that this relationship is very complex and should not be generalized (Celestina et al., 2019). The adding of “fresh biochar” to soil has been shown to induce short-term disturbance to the moisture equilibrium in soils due to the effect of capillary action, drawing moisture from soil pore spaces. In turn, this can cause negative effects on soil microbial communities and abundance through instantaneous desiccation (Dempster et al., 2012; Ameloot et al., 2013; Rex et al., 2015). However, soil pH and the other targeted traits were not significantly correlated with any bacterial diversity index.

## Conclusion

Wheat straw biochar better adapted the soil for durum wheat plant growth than woody biochar, strongly increasing soil EC and the concentrations of important ions (Na^+^, K^+^, and Cl^–^) available to plants. Biochar also enhanced plant growth, and the higher measured values of fresh and dry-weights, at both the above- and belowground levels, were for the samples treated with wheat straw biochar. From the rhizosphere microbiome analysis, it was evinced that untreated woody biochar led to a loss of bacterial strain richness, probably because of its nutrient deficiency, while more nutrient-rich biochars, such as wheat straw biochar and previously activated woody biochar, kept the bacterial alpha-diversity almost constant and stable. In addition, biochar showed a positive effect on the soil used, which was affected by light zinc and lead pollution, bringing a favorable decrease in the Zn available to plant, thus supporting its application in the remediation of soil with light contamination by heavy metals.

It is clear that, to attain the best advantages from the use of biochar, it is necessary to expend major efforts in the selection of the type of biochar used, with particular reference to the feedstock vegetal biomass and pyrolysis temperature, and the cultivar of the target crop to be cultivated in a specific agricultural soil. For durum wheat, in a low-organic matter soil, even with light heavy metal contamination, we suggest the combination of a straw-based-biochar with the Marco Aurelio variety. Of course, further comparative studies and, in particular, field trials have to be performed, aiming at choosing several pairs of biochar-cultivars that adapt and perform better in the various different wheat plantation regions.

## Data Availability Statement

The sequences have been submitted to the Sequence Read Archive (SRA) of the National Center for Biotechnology Information (NCBI) under the accession number PRJNA548437, referring to the BioProject entitled “Rhizospheric bacterial microbiota in durum wheat under biochar treatment.”

## Author Contributions

AL conceived the study and planned the experimental greenhouse setup, performed the experiments, wrote the manuscript original draft, reviewed, and edited the final version. GB performed the bioinformatic analysis of the bacterial rhizosphere microbiota, wrote the manuscript, and prepared the figures and tables related to the microbiome section. MT provided substantial help in the pot experiment, collaborated in the plant data collection, performed all experimental analyses related to soil and pore water chemical composition, and provided the tables containing all soil data. DM was responsible for the scanning electron microscopy analysis of the biochar samples and provided critical comments on the study. AB conceived and designed the experiments, supervised the analyses, interpreted the data, made a fundamental contribution to funding acquisition, drafted the manuscript, reviewed, and edited the final version. LT allowed the greenhouse experiment to be performed and provided important advice, particularly on the biochars and the low contaminated soil used in this work, obtained the funding for soil analysis, and made a fundamental contribution to the manuscript writing and to the whole revision. All authors read and approved the final manuscript.

## Conflict of Interest

The authors declare that the research was conducted in the absence of any commercial or financial relationships that could be construed as a potential conflict of interest.

## Footnotes

1 https://www.plant-phenotyping-network.eu/lw_resource/datapool/systemfiles/elements/files/e4061064-d535-11e8-8a88-dead53a91d31/current/document/bioadd-mad-it-eat.pdf

## Abbreviations

ASV: amplicon sequence variant
B1 −: untreated wood chip biochar
B1+: wood chip biochar activated by incubation with nutrient-rich solution
B2 −: untreated wheat straw biochar
C/N: carbon to nitrogen ration
CEC: cation exchange capacity
DW: dry weight
EC: electrical conductivity
FLL: flag leaf length
FW: fresh weight
mFLW: maximum flag leaf width
PH: plant height
SOM: soil organic matter
tAGFW and tAGDW: total aboveground fresh and dry weight
tBGFW and tBGDW: total belowground fresh and dry weight
TC: total carbon
TN: total nitrogen
TOC: total organic carbon
V1: Duilio durum wheat variety
V2: Marco Aurelio durum wheat variety
WHC: water holding capacity.

## References

[B1] Agegnehu G. Bass A. M. Nelson P. N. Bird M. I. (2016). Benefits of biochar, compost and biochar-compost for soil quality, maize yield and greenhouse gas emissions in a tropical agricultural soil. *Sci. Total. Environ.* 543 295–306. 10.1016/j.scitotenv.2015.11.054 26590867

[B2] Aguilar-Chávez Á Díaz-Rojas M. Cárdenas-Aquino M. D. Dendooven L. Luna-Guido M. (2012). Greenhouse gas emissions from a wastewater sludge-amended soil cultivated with wheat (*Triticum* spp. L.) as affected by different application rates of charcoal. *Soil Biol. Biochem.* 52 90–94. 10.1016/j.soilbio.2012.04.022

[B3] Albuquerque J. A. Salazar P. Barròn V. Torrent J. del Campillo M. C. Gallardo A. (2013). Enhanced wheat yield by biochar addition under different mineralization levels. *Agron. Sustain. Dev.* 33 475–484. 10.1007/s13593-012-0128-3

[B4] Ameloot N. De Neve S. Jegajeevagan K. Yildiz G. Buchan D. Funkuin Y. N. (2013). Short-term CO2 and N2O emissions and microbial properties of biochar amended sandy loam soils. *Soil Biol. Biochem.* 57 401–410. 10.1016/j.soilbio.2012.10.025

[B5] Atkinson C. J. Fitzgerald J. D. Hipps N. A. (2010). Potential mechanisms for achieving agricultural benefits from biochar application to temperate soil: a review. *Plant Soil* 337 1–18. 10.1007/s11104-010-0464-5

[B6] Bacci G. Ceccherini M. T. Bani A. Bazzicalupo M. Castaldini M. Galardini M. (2015). Exploring the dynamics of bacterial community composition in soil: the pan-bacteriome approach. *Antonie Van Leeuwenhoek* 107 785–797. 10.1007/s10482-014-0372-4 25563635

[B7] Bacci G. Cerri M. Lastrucci L. Ferranti F. Ferri V. Foggi B. (2018). Applying predictive models to decipher rhizobacterial modifications in common reed die-back affected populations. *Sci. Total. Environ.* 642 708–722. 10.1016/j.scitotenv.2018.06.066 29913366

[B8] Baronti S. Alberti G. Delle Vedove G. Di Gennaro F. Fellet G. Genesio L. (2010). The biochar option to improve plant yields: first results from some field and pot experiments in Italy. *Ital. J. Agron.* 5 3–11. 10.4081/ija.2010.3

[B9] Biederman L. A. Harpole W. S. (2013). Biochar and its effects on plant productivity and nutrient cycling: a meta-analysis. *GCB Bioener.* 5 202–214. 10.1111/gcbb.12037 31234136

[B10] Brennan A. Jiménez E. M. Puschenreiter M. Albuquerque J. A. Switzer C. (2014). Effects of biochar amendment on root traits and contaminant availability of maize plants in a copper and arsenic impacted soil. *Plant Soil* 379 351–360. 10.1007/s11104-014-2074-0

[B11] Bulgarelli D. Rott M. Schlaeppi K. Ver Loren van Themaat E. Ahmadinejad N. Assenza F. (2012). Revealing structure and assembly cues for *Arabidopsis* root-inhabiting bacterial microbiota. *Nature* 488 91–95. 10.1038/nature11336 22859207

[B12] Callahan B. J. McMurdie P. J. Rosen M. J. Han A. W. Johnson A. J. A. Holmes S. P. (2016). DADA2: high-resolution sample inference from Illumina amplicon data. *Nat. Methods* 13:581. 10.1038/nmeth.3869 27214047 PMC4927377

[B13] Carter M. R. Gregorich E. G. (2008). *Soil Sampling and Methods of Analysis. 2nd Edition. Canadian Society of Soil Science.* Boca Raton: CRC Press, 10.1201/9781420005271

[B14] Castaldi S. Riondino M. Baronti S. Esposito F. R. Marzaioli R. Rutigliano F. A. (2011). Impact of biochar application to a Mediterranean wheat crop on soil microbial activity and green house gas fluxes. *Chemosphere* 85 1464–1471. 10.1016/j.chemosphere.2011.08.031 21944041

[B15] Celestina C. Wood J. L. Manson J. B. Wang X. Sale P. W. G. Tang C. (2019). Microbial communities in top- and subsoil of repacked soil columns respond differently to amendments but their diversity is negatively correlated with plant productivity. *Sci. Rep.* 9:8890. 10.1038/s41598-019-45368-9 31222122 PMC6586782

[B16] Chen D. Guo H. Li R. Li L. Pan G. Chang A. (2016). Low uptake affinity cultivars with biochar to tackle Cd-tainted rice – A field study over four rice seasons in Hunan. *China Sci. Total Environ.* 541 1489–1498. 10.1016/j.scitotenv.2015.10.052 26490528

[B17] De Tender C. Haegeman A. Vandecasteele B. Clement L. Cremelie P. Dawyndt P. (2016). Dynamics in the strawberry rhizosphere microbiome in response to biochar and *Botrytis cinerea* leaf infection. *Front. Microbiol.* 7:2062 10.3389/fmicb.2016.02062 PMC5177642 28066380

[B18] Dempster D. N. Gleeson D. Solaiman Z. Jones D. L. Murphy D. (2012). Decreased soil microbial biomass and nitrogen mineralisation with Eucalyptus biochar addition to a coarse textured soil. *Plant Soil* 354 311–324. 10.1007/s11104-011-1067-5

[B19] Deng J. Yin Y. Zhu W. Zhou Y. (2018). Variations in soil bacterial community diversity and structures among different revegetation types in the Baishilazi Nature Reserve. *Front. Microbiol.* 9:2874. 10.3389/fmicb.2018.02874 30538689 PMC6277578

[B20] Di Cello F. Bevivino A. Chiarini L. Fani R. Paffetti D. Tabacchioni S. (1997). Biodiversity of a *Burkholderia cepacia* population isolated from the maize rhizosphere at different plant growth stages. *Appl. Environ. Microbiol.* 63 4485–4493. 9361434 10.1128/aem.63.11.4485-4493.1997 PMC168767

[B21] Elad Y. Cytryn E. Meller Harel Y. Lew B. Graber E. R. (2011). The biochar effect: plant resistance to biotic stresses. *Phytopathol. Mediterr.* 50 335–349. 10.14601/Phytopathol_Mediterr-9807 28270822

[B22] French E. Iyer-Pascuzzi A. S. (2018). A role for the gibberellins pathway in biochar-mediated growth promotion. *Sci. Rep.* 8:5389. 10.1038/s41598-018-23677-9 29599525 PMC5876386

[B23] Frenkel O. Jaiswal A. K. Elad Y. Lew B. Kammann C. Graber E. R. (2017). The effect of biochar on plant diseases: what should we learn while designing biochar substrates? *J. Environ. Eng. Landsc. Manag.* 25 105–113. 10.3846/16486897.2017.1307202

[B24] Han G. Lan J. Chen Q. Yu C. Bie S. (2017). Response of soil microbial community to application of biochar in cotton soils with different continuous cropping years. *Sci. Rep.* 7:101184. 10.1038/s41598-017-10427-6 28860603 PMC5578980

[B25] Hoagland D. R. Arnon D. I. eds. (1950). “The water-culture method for growing plants without soil,” in *Circular and California Agricultural Experiment Station* (Berkeley, CA: College of Agriculture, University of California), 32.

[B26] Jačka L. Trakal L. Ouřednìček P. Pohořelý M. Šìpek V. (2018). Biochar presence in soil significantly decreased saturated hydraulic conductivity due to swelling. *Soil Till. Res.* 184 181–185. 10.1016/j.still.2018.07.018

[B27] Jefferey S. Abalos D. Spokas K. Verheijen F. G. A. (2015). “Biochar effects on crop yield,” in *Biochar for environmental Management. Science, Technology and Implementation Chapter 12*, 2nd Edn, eds Lehmann J. Joseph S. (London: Routledge).

[B28] Jenkins J. R. Viger M. Arnold E. C. Harris Z. M. Ventura M. Miglietta F. (2017). Biochar alters the soil microbiome and soil function: results of next-generation amplicon sequencing across Europe. *GCB Bioenergy* 9 591–612. 10.1111/gcbb.12371

[B29] Kaetzl K. Lübken M. Gehring T. Wichern M. (2018). Efficient low-cost anaerobic treatment of wastewater using biochar and woodchip filters. *Water* 10:818 10.3390/w10070818

[B30] Kammann C. I. Schmidt H.-P. Messerchmidt N. Linsel S. Steffens D. Müller C. (2015). Plant growth improvement mediated by nitrate capture in co-composted biochar. *Sci. Rep.* 5:11080. 10.1038/srep11080 26057083 PMC4460888

[B31] Keiluweit M. Nico P. S. Johnson M. G. Kleber M. (2010). Dynamic molecular structure of plant biomass derived black carbon (biochar). *Environ. Sci. Technol.* 44 1247–1253. 10.1021/es9031419 20099810

[B32] Kelly C. N. Calderón F. C. Acosta-Martínez V. Mikha M. M. Benjamin J. Rutherford D. W. (2015). Switchgrass biochar effects on plant biomass and microbial dynamics in two soils from different regions. *Pedosphere* 25 329–342. 10.1016/S1002-0160(15)30001-1

[B33] Kiekens L. (1995). “Zinc,” in *Heavy Metals in Soils*, ed. Alloway B. J. (New York, NY: Springer US), 284–305.

[B34] Kim B.-R. Shin J. Guevarra R. B. Lee J. H. Kim D. W. Seol K.-H. (2017). Deciphering diversity indeces for a better understanding of microbial communities. *J. Microbiol. Biotechnol.* 27 2089–2093. 10.4014/jmb.1709.09027 29032640

[B35] Kolton M. Graber E. R. Tsehansky L. Elad Y. Cytryn E. (2017). Biochar-stimulated plant performance is strongly linked to microbial diversity and metabolic potential in the rhizosphere. *New Phytol.* 213 1393–1404. 10.1111/nph.14253 27780299

[B36] Kwak M.-J. Kong H. G. Choi K. Kwon S.-K. Song J. Y. Lee J. (2018). Rhizosphere microbiome structure alters to enable wilt resistance in tomato. *Nat. Biotechnol.* 36 1100–1109. 10.1038/nbt.4232 30295674

[B37] Lehmann J. (2007). Bio-energy in the black. *Front. Ecol. Environ.* 5:381–387. 10.1890/1540-9295(2007)5[381:BITB]2.0.CO;2

[B38] Lehmann J. Rillig M. C. Thies J. Masiello C. A. Hockaday W. C. Crowley D. (2011). Biochar effects on soil biota – a review. *Soil Biol. Biochem.* 43 1812–1836. 10.1016/j.soilbio.2011.04.022

[B39] Lei O. Zhang R. (2013). Effects of biochar derived from different feedstock and pyrolysis temperatures on soil, physical and hydraulic properties. *J Soils Sediments* 13 1561–1572. 10.1007/s11368-013-0738-7

[B40] Li Q. Lei Z. Song X. Zhang Z. Ying Y. Peng C. (2018). Biochar amendment decreases soil microbial biomass and increases bacterial diversity in Moso bamboo (*Phyllostachys edulis*) plantations under simulated nitrogen deposition. *Environ. Res. Lett.* 13:044029 10.1088/1748-9326/aab53a

[B41] Love M. I. Huber W. Anders S. (2014). Moderated estimation of fold change and dispersion for RNA-seq data with DESeq2. *Genom Biol.* 15:550. 10.1186/s13059-014-0550-8 25516281 PMC4302049

[B42] Mahoney A. K. Yin C. Hulbert S. H. (2017). Community structure, species variation, and potential functions of rhizosphere-associated bacteria of different winter wheat (*Triticum aestivum*) cultivars. *Front. Plant Sci.* 8:132. 10.3389/fpls.2017.00132 28243246 PMC5303725

[B43] Martin M. (2011). Cutadapt removes adapter sequences from high-throughput sequencing reads. *EMBnet J.* 17 10–12. 10.14806/ej.17.1.200

[B44] Mašek O. Buss W. Roy-Poirier A. Lowe W. Peters C. Brownsort P. (2018). Consistency of biochar properties over time and production scales: a characterisation of standard materials. *J. Anal. Appl. Pyrol.* 132 200–210. 10.1016/j.jaap.2018.02.020

[B45] Meng L. Sun T. Li M. Saleem M. Zhang Q. Wang C. (2019). Soil-applied biochar increases microbial diversity and wheat plant performance under herbicide fomesafen stress. *Ecotox Environ. Safe* 171 75–83. 10.1016/j.ecoenv.2018.12.065 30597319

[B46] Mollinedo J. Schumacher T. E. Chintala R. (2016). Biochar effects on phenotypic characteristics of “*wild*” and “*sickle*”. *Medicago truncatula* genotypes. *Plant Soil* 400 1–4. 10.1007/s11104-015-2708-x

[B47] Olmo M. K. Albuquerque J. A. Barròn V. del Campillo M. C. Gallardo A. Fuentes M. (2014). Wheat growth and yield responses to biochar addition under Mediterranean climate conditions. *Biol. Fertil. Soils* 50 1177–1187. 10.1007/s00374-014-0959-y

[B48] Palansooriya K. N. Wong J. T. F. Hashimoto Y. Huang L. Rinklebe J. Chang S. C. (2019). Response of microbial communities to biochar-amended soils: a critical review. *Biochar* 1 3–22. 10.1007/s42773-019-00009-2

[B49] Pandit N. R. Mulder J. Hale S. E. Martinsen V. Schmidt H. P. Cornelissen G. (2018). Biochar improves maize growth by alleviation of nutrient stress in a moderately acidic low-input Nepalese soil. *Sci. Total Environ.* 625 1380–1389. 10.1016/j.scitotenv.2018.01.022 29996435

[B50] Prajapati K. Modi H. A. (2012). The importance of potassium in plant growth – A review. *Indian J. Plant Sci.* 1 177–186.

[B51] Prendergast-Miller M. T. Duvall M. Sohi S. P. (2014). Biochar-root interactions are mediated by biochar nutrient content and impacts on soil nutrient availability. *Eur. J. Soil Sci.* 65 173–185. 10.1111/ejss.12079

[B52] Puga A. P. Abreu C. A. Melo L. C. A. Beesley L. (2015). Biochar application to a contaminated soil reduces the availability and plant uptake of zinc, lead and cadmium. *J. Environ. Manag.* 159 86–93. 10.1016/j.jenvman.2015.05.036 26048395

[B53] Qian X. Li H. Wang Y. Wu B. Wu M. Chen L. (2019). Leaf and root endosphere harbor lower fungal diversity and less complex fungal co-occurrence patterns than rhizosphere. *Front. Microbiol.* 10:1015. 10.3389/fmicb.2019.01015 31143169 PMC6521803

[B54] Qiao Q. Wang F. Zhang J. Chen Y. Zhang C. Liu G. (2017). Microbiome of cotton with soil type, genotype and developmental stage. *Sci. Rep.* 7:3940. 10.1038/s41598-017-04213-7 28638057 PMC5479781

[B55] Quast C. Pruesse E. Yilmaz P. Gerken J. Schweer T. Yarza P. (2012). The SILVA ribosomal RNA gene database project: improved data processing and web-based tools. *Nucleic Acids Res.* 41 D590–D596. 10.1093/nar/gks1219 23193283 PMC3531112

[B56] Quevauviller P. (1998). Operationally defined extraction procedures for soil and sediment analysis I. Standardization. *Trends Analyt. Chem.* 17 289–298. 10.1016/S0165-9936(97)00119-2

[B57] R Core Team (2018). *R: A Language and Environment for Statistical Computing.* Vienna: R Foundation for Statistical Computing.

[B58] Rex D. Schimmelpfenning S. Jansen-Willems A. Moser G. Kammann C. Müller C. (2015). Microbial community shifts 2.6 years after top dressing of Miscanthus biochar, hydrochar and feedstock on a temperate grassland site. *Plant Soil* 397 261–271. 10.1007/s11104-015-2618-y

[B59] Richter-Heitmann T. Eickhorst T. Knauth S. Friedrich M. W. Schmidt H. (2016). Evaluation of strategies to separate root-associated microbial communities: a crucial choice in rhizobiome research. *Front. Microbiol.* 7:773. 10.3389/fmicb.2016.00773 27252690 PMC4877504

[B60] Romkens P. F. A. M. Bouwman L. A. Boon G. T. (1999). Effect of plant growth on copper solubility and speciation in soil solution samples. *Environ. Pollut.* 106 315–321. 10.1016/S0269-7491(99)00106-2 15093027

[B61] Rutigliano F. A. Romano M. Marzaioli R. Baglivo I. Baronti S. Miglietta F. (2014). Effect of biochar addition on soil microbial community in a wheat crop. *Eur. J. Soil Biol.* 60 9–15. 10.1016/j.ejsobi.2013.10.007

[B62] Shade A. Handelsman J. (2012). Beyond the Venn diagram: the hunt for a core microbiome. *Environ. Microbiol.* 14 4–12. 10.1111/j.1462-2920.2011.02585.x 22004523

[B63] Shen Z. Zhang Y. Jin F. McMillan O. Al-Tabbaa A. (2017a). Qualitative and quantitative characterisation of adsorption mechanisms of lead on four biochars. *Sci. Total Environ.* 609 1401–1410. 10.1016/j.scitotenv.2017.08.008 28797146

[B64] Shen Z. Zhang Y. McMillan O. Jin F. Al-Tabbaa A. (2017b). Characteristics and mechanisms of nickel adsorption on biochars produced from wheat straw pellets and rice husk. *Environ. Sci. Pollut. Res.* 24 12809–12819. 10.1007/s11356-017-8847-2 28364204 PMC5418241

[B65] Smiley R. W. (1974). Rhizosphere pH as influenced by plants, soils and nitrogen fertilizers”. *Soil Sci. Soc. Am. J. Abstr.* 38 795–799. 10.2136/sssaj1974.03615995003800050030x

[B66] Solaiman Z. M. Blackwell P. Abbott L. K. Storer P. (2010). Direct and residual effect of biochar application on mycorrhizal root colonisation, growth and nutrition of wheat. *Aust. J. Soil Res.* 48 546–554. 10.1071/SR10002

[B67] Stephens M. (2016). False discovery rates: a new deal. *Biostatistics* 18:2. 10.1093/biostatistics/kxw041 27756721 PMC5379932

[B68] Sun H. Shi W. Zhou M. Ma X. Zhang H. (2019). Effect of biochar on nitrogen use efficiency, grain yield and amino acid content of wheat cultivated on saline soil. *Plant Soil Environ.* 65 83–89. 10.17221/525/2018-PSE

[B69] Takahashi S. Tomita J. Nishioka K. Hisada T. Nishijima M. (2014). Development of a prokaryotic universal primer for simultaneous analysis of Bacteria and Archea using next-generation sequencing. *PLoS One* 9:e105592. 10.1371/journal.pone.0105592 25144201 PMC4140814

[B70] Tammeorg P. Simojoki A. Mäkelä P. Stoddard F. L. Alakukku L. Helenius J. (2014). Biochar application to a fertile sandy clay loam in boreal conditions: effects on soil properties and yield formation of wheat, turnip rape and faba bean. *Plant Soil* 374 89–107. 10.1007/s11104-013-1851-5

[B71] Trakal L. Raya-Moreno I. Mitchell K. Beesley L. (2017). Stabilization of metal(loid)s in two contaminated agricultural soils: comparing biochar to its non-pyrolysed source material. *Chemosphere* 181 150–159. 10.1016/j.chemosphere.2017.04.064 28437740

[B72] UK Biochar Research Center (2014). *WSP700 Standard Biochar Specification Sheet. (–)Version 1.0.*

[B73] USDA (2011). *Carbon to Nitrogen Rations in Cropping Systems.* Greensboro, NC: NRCS East National Technology Support Center.

[B74] USDA Soil Survey Staff (2014). “Soil survey field and laboratory methods manual,” in *Soil Survey Investigations Report No. 51, Version 2.0*, eds Burt R. and Soil Survey Staff, (Washington, DC: U.S. Department of Agriculture).

[B75] Vaccari F. P. Baronti S. Lugato E. Genesio L. Castaldi S. Fornasier F. (2011). Biochar as a strategy to sequester carbon and increase yield in durum wheat. *Eur. J. Agron.* 34 231–238. 10.1016/j.eja.2011.01.006

[B76] Wan X. H. Huang Z. Q. He Z. M. Yu Z. P. Wang M. H. Davis M. R. (2015). Soil C:N ratio is the major determinant of soil microbial community structure in subtropical coniferous and broadleaf forest plantations. *Plant Soil* 387 103–116. 10.1007/s11104-014-2277-4

[B77] Wang R. Wei S. Jia P. Liu T. Hou D. Xie R. (2019). Biochar significantly alters rhizobacterial communities and reduces Cd concentration in rice grains grown on Cd-contaminated soils. *Sci. Total Environ.* 676 627–638. 10.1016/j.scitotenv.2019.04.133 31051368

[B78] Wang Y. Hu Y. Zhao X. Wang S. Xing G. (2013). Comparisons of biochar properties from wood material and crop residues at different temperatures and residence times. *Energy Fuels* 27 5890–5899. 10.1021/ef400972z

[B79] Wiedner K. Rumpel C. Steiner C. Pozzi A. Maas R. Glaser B. (2013). Chemical evaluation of chars produced by thermochemical conversion (gasification, pyrolysis and hydrothermal carbonization) of agro-industrial biomass on a commercial scale. *Biomass Bioenergy* 59 264–278. 10.1016/j.biombioe.2013.08.026

[B80] Xiang Y. Deng Q. Duan H. Guo Y. (2017). Effects of biochar application on root traits: a meta-analysis. *GCB Bioenergy* 9 1563–1572. 10.1111/gcbb.12449

[B81] Zama E. F. Reid B. J. Arp H. P. H. Sun G. X. Yuan H. Y. Zhu Y. G. (2018). Advances in research on the use of biochar in soil for remediation: a review. *J. Soils Sediments* 18:2433 10.1007/s11368-018-2000-9

[B82] Zemanová V. Trakal L. Ochecová P. Szaková J. Pavliková D. (2014). A model experiment: competitive sorption of Cd, Cu, Pb and Zn by three different soils. *Soil Water Res.* 9 97–103. 10.17221/50/2013-SWR

[B83] Zhang H. Chen C. Gray E. M. Boyd S. E. (2017). Effect of feedstock and pyrolysis temperature on properties of biochar governing end use efficacy. *Biomass Bioenergy* 105 136–146. 10.1016/j.biombioe.2017.06.024

[B84] Zhang X. Wang H. He L. Lu K. Sarmah A. Li J. (2013). Using biochar for remediation of soils contaminated with heavy metals and organic pollutants. *Environ. Sci. Pollut. Res.* 20 8472–8483. 10.1007/s11356-013-1659-0 23589248

