# Supplementary material for: The Impact of Soil-Applied Biochars From Different Vegetal Feedstocks on Durum Wheat Plant Performance and Rhizospheric Bacterial Microbiota in Low Metal-Contaminated Soil
Source: Front Microbiol. 2019 Dec 10;10:2694. doi: 10.3389/fmicb.2019.02694 (PMC6916200; doi:10.3389/fmicb.2019.02694)
Supplement: Supplementary file 1 [file Data_Sheet_1.zip › Supplementary_Material_1_Latini_et_al.docx]

Supplementary Material 1

**Table S1.** Characteristics of the studied biochars

|  | | | | | |
| --- | --- | --- | --- | --- | --- |
| **GENERAL INFORMATION** | | | | | |
|  | | **B1** | | **B2** | |
|  | |  | |  | |
| Provider | Carbon Terra GmbH (Germany); *www.carbon-terra.eu* | | UK Biochar Research Center (University of Edinburgh, UK); *www.biochar.ar.uk* | |  |
|  |  | |  | |  |
| Feedstock | Wood chips (80% coniferous, 20% deciduous wood)^a^ | | Wheat straw pellets | |  |
|  |  | |  | |  |
| Production | Continuous pyrolysis process in vertical rotors (Schottdorf type reactor) with pyrolysis treatment temperature approx. 700°C for about 36 h^a^ | | Pilot-scale rotator kiln pyrolysis unit, nominal peak temperature 700°C^b^; then oven drying at 60°C for 48 h^c^ | |  |
|  | |  | |  | |
|  | | | | | |
| **PHYSICOCHEMICAL PROPERTIES** | | | | | |
|  | | **B1** | | **B2** | |
|  | |  | |  | |
| Specific surface area (BET) | 143.8 (m^2^ g^-1^ in fresh matter)^a^; 485 (m^2^ g^-1^)^d^; 1.11·10^8^ (m^2^ m^-3^)^d^ | | 23.20 (m^2^ g^-1^)^c^ | |  |
|  |  | |  | |  |
| pH | 9.5 (in fresh matter)^a^; 9.78 (in H_2_O)^e^; 8.56 (in CaCl_2_)^e^ | | 10.3 ± 0.19 (4)^b^ | |  |
|  |  | |  | |  |
| EC | 578 (μS cm^-1^ in fresh matter)^a^; 469 (μS cm^-1^)^e^ | | 1520 ± 420 (4) (μS cm^-1^)^b^ | |  |
|  |  | |  | |  |
| CEC | N.A. | | 12.50 (cmol/Kg)^f^ | |  |
|  |  | |  | |  |
| Ash content | 14.1 (wt% in fresh matter at 550°C)^a^; 16.3 (wt% in dry matter at 550°C)^a^; 23.5 (wt%)^e^ | | 23.82 ± 2.33 (5) (wt%)^b^ | |  |
|  |  | |  | |  |
| TC | 65.7 (wt% in fresh matter)^a^; 75.8 (wt% in dry matter)^a^; 89.2 (wt%)^d^ | | 69.04 ± 1.34 (4) (wt%)^b^ | |  |
|  |  | |  | |  |
| TOC | 65.2 (wt% in fresh matter)^a^; 75.3 (wt% in dry matter)^a^; 67.4 (wt%)^e^ | | N.A. | |  |
|  |  | |  | |  |
| TN | 0.38 (wt% in fresh matter)^a^; 0.43 (wt% in dry matter)^a^; 1.0 (wt%)^d^; 0,63 wt%)^e^ | | 1.32 ± 0.03 (4) (wt%)^b^ | |  |
|  |  | |  | |  |
| Phosphorous (P) | 810 (mg/Kg in dry matter)^a^; 4300 (mg Kg^-1^)^d^ | | 2500 mg/Kg^c^ | |  |
|  |  | |  | |  |
| Hydrogen (H) | 1.7 (wt%)^e^; 1.13 (wt% in fresh matter)^a^; 1.31 (wt% in dry matter)^a^; 1.6 (wt%)^d^ | | 1.18 ± 0.04 (4) (wt%)^b^ | |  |
|  |  | |  | |  |
| Oxygen (O) | 6.3 (wt%)^e^; 5.3 (wt% in fresh matter)^a^; 6.2 (wt% in dry matter)^a^; 1.9 (wt%)^d^ | | 5.30 ± 1.06 (4) (wt%)^b^ | |  |
|  |  | |  | |  |
| C/N ratio | 107.0^e^; 89.2^d^ | | N.A. | |  |

*EC is electrical conductivity; CEC is cation exchange capacity; TC is total (organic plus inorganic) carbon; TOC is total organic carbon; TN is total nitrogen; N.A. is not available; bdl is below detection limit; ^a^Kammann et al. 2015 (pyrolisys temperature about 700°C); ^b^UK Biochar Research Centre 2014; ^c^Shen et al. 2017b; ^d^Kaetzl et al. 2018; ^e^Wiedner et al. 2013 (pyrolisys temperature about 550°C); ^f^Shen et al. 2017a.*
