# Supplementary material for: The Impact of Soil-Applied Biochars From Different Vegetal Feedstocks on Durum Wheat Plant Performance and Rhizospheric Bacterial Microbiota in Low Metal-Contaminated Soil
Source: Front Microbiol. 2019 Dec 10;10:2694. doi: 10.3389/fmicb.2019.02694 (PMC6916200; doi:10.3389/fmicb.2019.02694)
Supplement: Supplementary file 1 [file Data_Sheet_1.zip › Supplementary_Material_11_Latini_et_al.docx]

Supplementary Material 11

**Table S9.** Pearson’s correlation coefficients between soil rhizosphere bacterial diversity indexes (Shannon and Inverse Simpson) and soil chemical properties

| Indexes | pH(H_2_O) | pH(KCl) | EC | CEC | TC | TOC | TN | C/N |
| --- | --- | --- | --- | --- | --- | --- | --- | --- |
| Shannon | 0.572^0.139^ | 0.346^0.401^ | 0.116^0.784^ | 0.416^0.305^ | -0.735^0.038^* | -0.561^0.148^ | -0.697^0.055^ | -0.611^0.107^ |
| Inverse Simpson | 0.600^0.115^ | 0.381^0.352^ | 0.371^0.366^ | 0.460^0.251^ | -0.801^0.017^* | -0.672^0.068^ | -0.755^0.030^* | -0.628^0.095^ |

*Significance levels (p < 0.05) are indicated by superscript.*
