# Supplementary material for: The Impact of Soil-Applied Biochars From Different Vegetal Feedstocks on Durum Wheat Plant Performance and Rhizospheric Bacterial Microbiota in Low Metal-Contaminated Soil
Source: Front Microbiol. 2019 Dec 10;10:2694. doi: 10.3389/fmicb.2019.02694 (PMC6916200; doi:10.3389/fmicb.2019.02694)
Supplement: Supplementary file 1 [file Data_Sheet_1.zip › Supplementary_Material_2_Latini_et_al.docx]

Supplementary Material 2

**Table S2.** Main characteristics of the two durum wheat varieties used in the current experiment

| **Characteristics** |  | **Duilio** | **Marco Aurelio** |
| --- | --- | --- | --- |
| Morpho-physiological traits | Spiking period  Size | Early  Medium-low | Medium  Medium |
| Resistance | Lodging  Winter cold | Medium  Medium | Good  Good |
| Tolerance | Powdery mildew  Brown rust  Septoria  *Fusarium* spp. | Medium  Good  Medium  Medium | Good  Good  Optimal  Good |
| Qualitative traits | 1000 g weight  Hectolitric weight  Yellow index  Protein content  Gluten index | 47-52 g  Good  Medium  Medium  Medium | 53-58 g  Good  Optimal  Excellent  Optimal |

*Information from www.sisonweb.com*
