# Supplementary material for: The Impact of Soil-Applied Biochars From Different Vegetal Feedstocks on Durum Wheat Plant Performance and Rhizospheric Bacterial Microbiota in Low Metal-Contaminated Soil
Source: Front Microbiol. 2019 Dec 10;10:2694. doi: 10.3389/fmicb.2019.02694 (PMC6916200; doi:10.3389/fmicb.2019.02694)
Supplement: Supplementary file 1 [file Data_Sheet_1.zip › Supplementary_Material_3_Latini_et_al.docx]

Supplementary Material 3

# Plant irrigation

During the experiment set up, pots were initially filled with 700 g of medium. In each pot, in order to keep soil moisture constant along the entire experiment duration, in particular around the plantlet during its early development, we used an absorbent string and a 15 ml Falcon tube containing 15 ml water (Fig. 1A, B). The string was laid on the soil pot surface in a circle fashion, with one of its extremities passing through a hole, previously made through the pot and than through the cap of the tube, and arriving up to the bottom part of the tube. After plantlet transplantation, we put more medium over pots, until the seed coat and the string were around 1 cm below the soil surface.


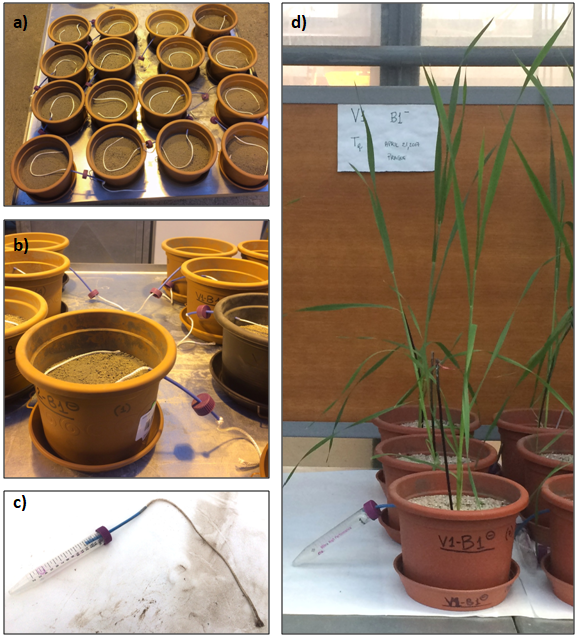


**Figure S1.** **System used for keeping wet the upper part of the soil pot and maintaining an almost constant soil water holding capacity. a)**, **b)** Pictures showing the way the absorbent string was laid on the pot soil surfaces and connected to a 15 ml Falcon tube cap; **c)** the Falcon tube is filled with water, for osmosis water goes from the tube to the soil in order to keep soil moisture level constant, particularly at the beginning of the experiment when the seedlings have been just transplanted into the pots and are very small; **d)** one sample pot at the end of the experiment (T_f_), with the Falcon tube that provides water to the soil in order to minimize possible dehydration that may occur in the time between two irrigations (plants received ~ 300 ml water three times per week).
