# Supplementary material for: The Impact of Soil-Applied Biochars From Different Vegetal Feedstocks on Durum Wheat Plant Performance and Rhizospheric Bacterial Microbiota in Low Metal-Contaminated Soil
Source: Front Microbiol. 2019 Dec 10;10:2694. doi: 10.3389/fmicb.2019.02694 (PMC6916200; doi:10.3389/fmicb.2019.02694)
Supplement: Supplementary file 1 [file Data_Sheet_1.zip › Supplementary_Material_4_Latini_et_al.docx]

Supplementary Material 4

**Table S3.** Concentrations (*ng/μl*) of the soil gDNA at Qubit 4 Fluorometer

|  | **1^st^ read** | **2^nd^ read** | **3^rd^ read** | **4^th^ read** | **Average *ng/μl*** | **Std.Dv** |
| --- | --- | --- | --- | --- | --- | --- |
|  | *ng/μl* | *ng/μl* | *ng/μl* | *ng/μl* | *ng/μl* | *ng/μl* |
| **V1 C- P1_A** | 78,40 | 74,60 | 73,80 | 76,20 | 75,75 | 2,03 |
| **V1 C- P2_A** | 90,00 | 92,40 | 87,60 | 92,00 | 90,50 | 2,20 |
| **V1 C- P3_A** | N.A. | N.A. | N.A. | N.A. | N.A. | N.A. |
| **V1 C- P4_A** | 81,00 | 81,40 | 80,00 | 80,80 | 80,80 | 0,59 |
| **V1 C- P5_A** | 83,00 | 81,00 | 81,20 | 82,20 | 81,85 | 0,93 |
| **V1 C- P6_A** | 64,20 | 64,20 | 62,40 | 63,00 | 63,45 | 0,90 |
| **V1 C- P1_B** | 50,40 | 45,20 | 45,60 | 49,40 | 47,65 | 2,64 |
| **V1 C- P2_B** | 3,94 | 2,90 | 3,30 | 4,04 | 3,55 | 0,54 |
| **V1 C- P3_B** | N.A. | N.A. | N.A. | N.A. | N.A. | N.A. |
| **V1 C- P4_B** | 9,66 | 8,52 | 8,92 | 9,94 | 9,26 | 0,65 |
| **V1 C- P5_B** | 10,20 | 9,64 | 9,10 | 10,40 | 9,84 | 0,59 |
| **V1 C- P6_B** | 8,96 | 7,90 | 7,80 | 8,56 | 8,31 | 0,55 |
| **V1 B1- P1_A** | 65,40 | 66,80 | 65,40 | 65,60 | 65,80 | 0,67 |
| **V1 B1- P2_A** | 79,60 | 79,60 | 78,60 | 79,20 | 79,25 | 0,47 |
| **V1 B1- P3_A** | 80,60 | 81,20 | 80,20 | 80,20 | 80,55 | 0,47 |
| **V1 B1- P4_A** | 97,80 | 97,80 | 92,00 | 93,60 | 95,30 | 2,96 |
| **V1 B1- P5_A** | 99,80 | 99,80 | 97,00 | 96,00 | 98,15 | 1,95 |
| **V1 B1- P6_A** | 89,20 | 88,00 | 87,40 | 86,20 | 87,70 | 1,25 |
| **V1 B1- P1_B** | 73,10 | 75,20 | 85,20 | 78,60 | 78,03 | 5,29 |
| **V1 B1- P2_B** | 12,60 | 12,20 | 12,30 | 12,30 | 12,35 | 0,17 |
| **V1 B1- P3_B** | 7,84 | 7,72 | 7,52 | 7,54 | 7,66 | 0,15 |
| **V1 B1- P4_B** | 35,60 | 35,20 | 34,80 | 35,20 | 35,20 | 0,33 |
| **V1 B1- P5_B** | 6,18 | 6,22 | 5,96 | 5,80 | 6,04 | 0,20 |
| **V1 B1- P6_B** | 63,00 | 61,20 | 60,00 | 59,40 | 60,90 | 1,59 |
| **V1 B1+ P1_A** | 168,40 | 180,60 | 178,20 | 177,40 | 176,15 | 5,34 |
| **V1 B1+ P2_A** | 155,20 | 161,80 | 161,20 | 160,00 | 159,55 | 2,99 |
| **V1 B1+ P3_A** | 202,00 | 212,00 | 195,80 | 206,00 | 203,95 | 6,81 |
| **V1 B1+ P4_A** | 179,20 | 191,20 | 189,60 | 185,60 | 186,40 | 5,35 |
| **V1 B1+ P5_A** | 234,00 | 230,00 | 228,00 | 228,00 | 230,00 | 2,83 |
| **V1 B1+ P6_A** | 214,00 | 212,00 | 212,00 | 208,00 | 211,50 | 2,52 |
| **V1 B1+ P1_B** | 197,80 | 186,00 | 191,80 | 188,80 | 191,10 | 5,06 |
| **V1 B1+ P2_B** | 105,60 | 101,80 | 101,80 | 102,00 | 102,80 | 1,87 |
| **V1 B1+ P3_B** | 94,60 | 92,20 | 93,20 | 90,60 | 92,65 | 1,68 |
| **V1 B1+ P4_B** | 181,80 | 188,40 | 186,40 | 186,80 | 185,85 | 2,83 |
| **V1 B1+ P5_B** | 198,40 | 196,60 | 194,60 | 195,00 | 196,15 | 1,73 |
| **V1 B1+ P6_B** | 162,40 | 156,80 | 156,00 | 155,60 | 157,70 | 3,17 |
| **V1 B2- P1_A** | 123,00 | 124,80 | 124,60 | 123,20 | 123,90 | 0,93 |
| **V1 B2- P2_A** | 167,20 | 167,60 | 168,80 | 169,20 | 168,20 | 0,95 |
| **V1 B2- P3_A** | 107,20 | 108,20 | 107,20 | 107,20 | 107,45 | 0,50 |
| **V1 B2- P4_A** | 92,00 | 95,00 | 95,20 | 94,20 | 94,10 | 1,47 |
| **V1 B2- P5_A** | 134,20 | 136,80 | 134,80 | 135,20 | 135,25 | 1,11 |
| **V1 B2- P6_A** | 117,80 | 120,80 | 122,00 | 122,60 | 120,80 | 2,14 |
| **V1 B2- P1_B** | 139,20 | 142,00 | 142,20 | 140,40 | 140,95 | 1,42 |
| **V1 B2- P2_B** | 163,80 | 163,60 | 155,00 | 148,60 | 157,75 | 7,35 |
| **V1 B2- P3_B** | 107,60 | 108,80 | 105,40 | 107,00 | 107,20 | 1,41 |
| **V1 B2- P4_B** | 80,80 | 82,80 | 83,00 | 82,20 | 82,20 | 0,99 |
| **V1 B2- P5_B** | 126,00 | 128,00 | 127,60 | 126,40 | 127,00 | 0,95 |
| **V1 B2- P6_B** | 137,80 | 135,80 | 138,40 | 135,40 | 136,85 | 1,47 |
| **V2 C- P1_A** | 181,20 | 193,80 | 202,00 | 187,20 | 191,05 | 8,93 |
| **V2 C- P2_A** | 330,00 | 340,00 | 348,00 | 328,00 | 336,50 | 9,29 |
| **V2 C- P3_A** | 290,00 | 302,00 | 316,00 | 292,00 | 300,00 | 11,89 |
| **V2 C- P4_A** | 284,00 | 290,00 | 302,00 | 288,00 | 291,00 | 7,75 |
| **V2 C- P5_A** | 238,00 | 238,00 | 244,00 | 236,00 | 239,00 | 3,46 |
| **V2 C- P6_A** | 254,00 | 256,00 | 260,00 | 250,00 | 255,00 | 4,16 |
| **V2 C- P1_B** | 230,00 | 232,00 | 248,00 | 232,00 | 235,50 | 8,39 |
| **V2 C- P2_B** | 234,00 | 232,00 | 246,00 | 236,00 | 237,00 | 6,22 |
| **V2 C- P3_B** | 286,00 | 290,00 | 310,00 | 294,00 | 295,00 | 10,52 |
| **V2 C- P4_B** | 290,00 | 284,00 | 306,00 | 290,00 | 292,50 | 9,43 |
| **V2 C- P5_B** | 272,00 | 272,00 | 292,00 | 282,00 | 279,50 | 9,57 |
| **V2 C- P6_B** | 220,00 | 216,00 | 234,00 | 224,00 | 223,50 | 7,72 |
| **V2 B1- P1_A** | 256,00 | 256,00 | 262,00 | 254,00 | 257,00 | 3,46 |
| **V2 B1- P2_A** | N.A. | N.A. | N.A. | N.A. | N.A. | N.A. |
| **V2 B1- P3_A** | 169,20 | 167,80 | 177,20 | 172,80 | 171,75 | 4,20 |
| **V2 B1- P4_A** | 270,00 | 268,00 | 280,00 | 270,00 | 272,00 | 5,42 |
| **V2 B1- P5_A** | 140,40 | 143,40 | 150,20 | 146,80 | 145,20 | 4,24 |
| **V2 B1- P6_A** | 226,00 | 234,00 | 246,00 | 240,00 | 236,50 | 8,54 |
| **V2 B1- P1_B** | 252,00 | 254,00 | 260,00 | 246,00 | 253,00 | 5,77 |
| **V2 B1- P2_B** | N.A. | N.A. | N.A. | N.A. | N.A. | N.A. |
| **V2 B1- P3_B** | 190,80 | 191,20 | 197,80 | 192,40 | 193,05 | 3,24 |
| **V2 B1- P4_B** | 206,00 | 208,00 | 216,00 | 210,00 | 210,00 | 4,32 |
| **V2 B1- P5_B** | 195,20 | 197,60 | 206,00 | 199,40 | 199,55 | 4,63 |
| **V2 B1- P6_B** | 264,00 | 270,00 | 284,00 | 276,00 | 273,50 | 8,54 |
| **V2 B1+ P1_A** | 206,00 | 210,00 | 222,00 | 228,00 | 216,50 | 10,25 |
| **V2 B1+ P2_A** | N.A. | N.A. | N.A. | N.A. | N.A. | N.A. |
| **V2 B1+ P3_A** | 268,00 | 270,00 | 280,00 | 286,00 | 276,00 | 8,49 |
| **V2 B1+ P4_A** | 258,00 | 268,00 | 278,00 | 284,00 | 272,00 | 11,43 |
| **V2 B1+ P5_A** | 190,80 | 204,00 | 214,00 | 216,00 | 206,20 | 11,53 |
| **V2 B1+ P6_A** | 216,00 | 218,00 | 222,00 | 224,00 | 220,00 | 3,65 |
| **V2 B1+ P1_B** | 248,00 | 256,00 | 268,00 | 276,00 | 262,00 | 12,44 |
| **V2 B1+ P2_B** | N.A. | N.A. | N.A. | N.A. | N.A. | N.A. |
| **V2 B1+ P3_B** | 274,00 | 278,00 | 290,00 | 300,00 | 285,50 | 11,82 |
| **V2 B1+ P4_B** | 208,00 | 218,00 | 226,00 | 232,00 | 221,00 | 10,39 |
| **V2 B1+ P5_B** | 204,00 | 214,00 | 222,00 | 224,00 | 216,00 | 9,09 |
| **V2 B1+ P6_B** | 188,20 | 195,80 | 200,00 | 204,00 | 197,00 | 6,75 |
| **V2 B2- P1_A** | 181,40 | 182,00 | 186,60 | 189,00 | 184,75 | 3,66 |
| **V2 B2- P2_A** | N.A. | N.A. | N.A. | N.A. | N.A. | N.A. |
| **V2 B2- P3_A** | 232,00 | 228,00 | 236,00 | 236,00 | 233,00 | 3,83 |
| **V2 B2- P4_A** | 228,00 | 228,00 | 228,00 | 234,00 | 229,50 | 3,00 |
| **V2 B2- P5_A** | 200,00 | 196,20 | 199,80 | 202,00 | 199,50 | 2,41 |
| **V2 B2- P6_A** | 204,00 | 206,00 | 206,00 | 208,00 | 206,00 | 1,63 |
| **V2 B2- P1_B** | 186,00 | 191,00 | 196,80 | 200,00 | 193,45 | 6,21 |
| **V2 B2- P2_B** | N.A. | N.A. | N.A. | N.A. | N.A. | N.A. |
| **V2 B2- P3_B** | 232,00 | 236,00 | 224,00 | 228,00 | 230,00 | 5,16 |
| **V2 B2- P4_B** | 224,00 | 236,00 | 244,00 | 244,00 | 237,00 | 9,45 |
| **V2 B2- P5_B** | 286,00 | 294,00 | 300,00 | 298,00 | 294,50 | 6,19 |
| **V2 B2- P6_B** | 222,00 | 226,00 | 228,00 | 232,00 | 227,00 | 4,16 |

Two independent DNA extractions (A and B) have been carried out for each collected rhizosphere sample, and then pooled together in equimolar ratio. Each DNA extraction has been read four time at the fluorometer and the proceeding average value has been used for preparing the final samples for the sequencing.

P1, P2, …, P6 indicate the number of the plant biological replica. N.A. stands for not available DNA sample, and generally refers to sample plants not grown enough or showing some problems. Very low concentrated samples (reported in blue colour) were not used for the further analysis.
