# Supplementary material for: The Impact of Soil-Applied Biochars From Different Vegetal Feedstocks on Durum Wheat Plant Performance and Rhizospheric Bacterial Microbiota in Low Metal-Contaminated Soil
Source: Front Microbiol. 2019 Dec 10;10:2694. doi: 10.3389/fmicb.2019.02694 (PMC6916200; doi:10.3389/fmicb.2019.02694)
Supplement: Supplementary file 1 [file Data_Sheet_1.zip › Supplementary_Material_5_Latini_et_al.docx]

Supplementary Material 5

**Table S4.** Number of reads retained at each clustering step

|  | **raw_**  **reads** | **no_**  **adapt** | **quality_**  **filtered** | **merged_and_**  **denoised** | **non_**  **chimeric** | **bacteria** | **no_**  **chloroplast** | **no_**  **mitochondria** |
| --- | --- | --- | --- | --- | --- | --- | --- | --- |
| **V1 C- P2** | 135780 | 134861 | 96942 | 67397 | 64798 | 63305 | 62951 | 54833 |
| **V1 C- P4** | 168899 | 167772 | 113841 | 78278 | 75533 | 73987 | 73209 | 62849 |
| **V1 C- P5** | 95991 | 95302 | 69359 | 47802 | 46385 | 45784 | 45476 | 40070 |
| **V1 C- P6** | 108809 | 108097 | 79275 | 55198 | 53503 | 52913 | 52587 | 43687 |
| **V1 B1- P1** | 37197 | 36947 | 27204 | 20579 | 19508 | 19447 | 19330 | 17969 |
| **V1 B1- P3** | 47167 | 46832 | 33089 | 22904 | 22461 | 22296 | 22149 | 19317 |
| **V1 B1- P4** | 79328 | 78797 | 57466 | 43388 | 42004 | 41839 | 41514 | 36115 |
| **V1 B1- P5** | 128060 | 127191 | 92130 | 69792 | 67534 | 66978 | 66534 | 55692 |
| **V1 B1+ P2** | 101766 | 101075 | 72908 | 49410 | 47877 | 46881 | 46596 | 37635 |
| **V1 B1+ P3** | 126722 | 125914 | 89857 | 62189 | 60232 | 59508 | 59194 | 49138 |
| **V1 B1+ P4** | 100204 | 99429 | 72941 | 47304 | 45472 | 44417 | 44127 | 37776 |
| **V1 B1+ P5** | 123275 | 122481 | 90114 | 62390 | 60544 | 59241 | 58454 | 49118 |
| **V1 B1+ P6** | 140106 | 139208 | 102387 | 75199 | 72467 | 70653 | 70093 | 59091 |
| **V1 B2- P1** | 81249 | 80552 | 57661 | 40967 | 40049 | 39704 | 39489 | 35440 |
| **V1 B2- P2** | 116582 | 115754 | 86015 | 67489 | 65493 | 65317 | 64744 | 57826 |
| **V1 B2- P3** | 30770 | 30447 | 22411 | 18744 | 17825 | 17796 | 17764 | 17465 |
| **V1 B2- P4** | 90105 | 89463 | 65583 | 49563 | 48173 | 47952 | 47627 | 41747 |
| **V2 C- P1** | 107529 | 106744 | 78194 | 55837 | 54079 | 53722 | 53299 | 46992 |
| **V2 C- P2** | 54963 | 54640 | 40012 | 26870 | 26468 | 26313 | 26087 | 23366 |
| **V2 C- P4** | 6372 | 6292 | 4658 | 4264 | 4194 | 4194 | 4190 | 4190 |
| **V2 C- P6** | 103374 | 102809 | 75316 | 48647 | 46954 | 46323 | 46013 | 39179 |
| **V2 B1- P3** | 35602 | 35357 | 24910 | 16010 | 15734 | 15643 | 15525 | 13490 |
| **V2 B1- P4_OTU1** | 53841 | 53462 | 35824 | 22097 | 21538 | 21495 | 21402 | 18491 |
| **V2 B1- P5** | 73549 | 73099 | 53443 | 36971 | 35952 | 35739 | 35502 | 30814 |
| **V2 B1+ P1** | 37822 | 37282 | 17680 | 6315 | 5841 | 5712 | 5712 | 5395 |
| **V2 B1+ P3** | 47373 | 47077 | 34065 | 24004 | 23664 | 23526 | 23362 | 21168 |
| **V2 B1+ P4** | 78404 | 77933 | 57072 | 36077 | 35051 | 34627 | 34387 | 30114 |
| **V2 B1+ P5** | 68549 | 68203 | 50296 | 33205 | 32516 | 32308 | 31967 | 26917 |
| **V2 B2- P1** | 75753 | 75376 | 54967 | 33927 | 32941 | 32852 | 32699 | 28862 |
| **V2 B2- P3** | 33273 | 33074 | 23757 | 13575 | 13198 | 13136 | 13075 | 11552 |
| **V2 B2- P4** | 76387 | 75928 | 54590 | 35494 | 34583 | 34370 | 34194 | 29351 |
| **V2 B2- P5** | 92363 | 91774 | 67483 | 45987 | 44878 | 44538 | 44233 | 38432 |
| **V2 B2- P6** | 89480 | 88944 | 64769 | 43758 | 42788 | 42511 | 42212 | 37714 |
| **V2 B1- P4_OTU2** | 107818 | 107259 | 78031 | 52751 | 50621 | 50376 | 50098 | 42494 |
| **V2 B1- P4_OTU3** | 90877 | 90259 | 64751 | 43971 | 42616 | 42150 | 41897 | 35588 |
| **V1 B1- P2** | 38262 | 37890 | 28557 | 21594 | 21226 | 21148 | 21081 | 19350 |
| **V1 B1- P6** | 8758 | 8613 | 6410 | 5258 | 4797 | 4793 | 4793 | 4784 |
| **V1 B2- P6** | 61668 | 61234 | 48560 | 38838 | 37543 | 37454 | 37247 | 35068 |
| **V2 B1- P1** | 6102 | 6005 | 4654 | 4414 | 4365 | 4365 | 4359 | 4359 |
| **V2 B1+ P6** | 12145 | 11863 | 9786 | 8084 | 6905 | 6905 | 6905 | 6905 |
| **V1 C- P2** | 135780 | 134861 | 96942 | 67397 | 64798 | 63305 | 62951 | 54833 |
| **V1 C- P4** | 168899 | 167772 | 113841 | 78278 | 75533 | 73987 | 73209 | 62849 |
| **V1 C- P5** | 95991 | 95302 | 69359 | 47802 | 46385 | 45784 | 45476 | 40070 |
| **V1 C- P6** | 108809 | 108097 | 79275 | 55198 | 53503 | 52913 | 52587 | 43687 |
| **V1 B1- P1** | 37197 | 36947 | 27204 | 20579 | 19508 | 19447 | 19330 | 17969 |

V2B2- P4 samples labeled with "OTU1-2-3" were run and used to choose the data filtering cut-off before downstream analysis.


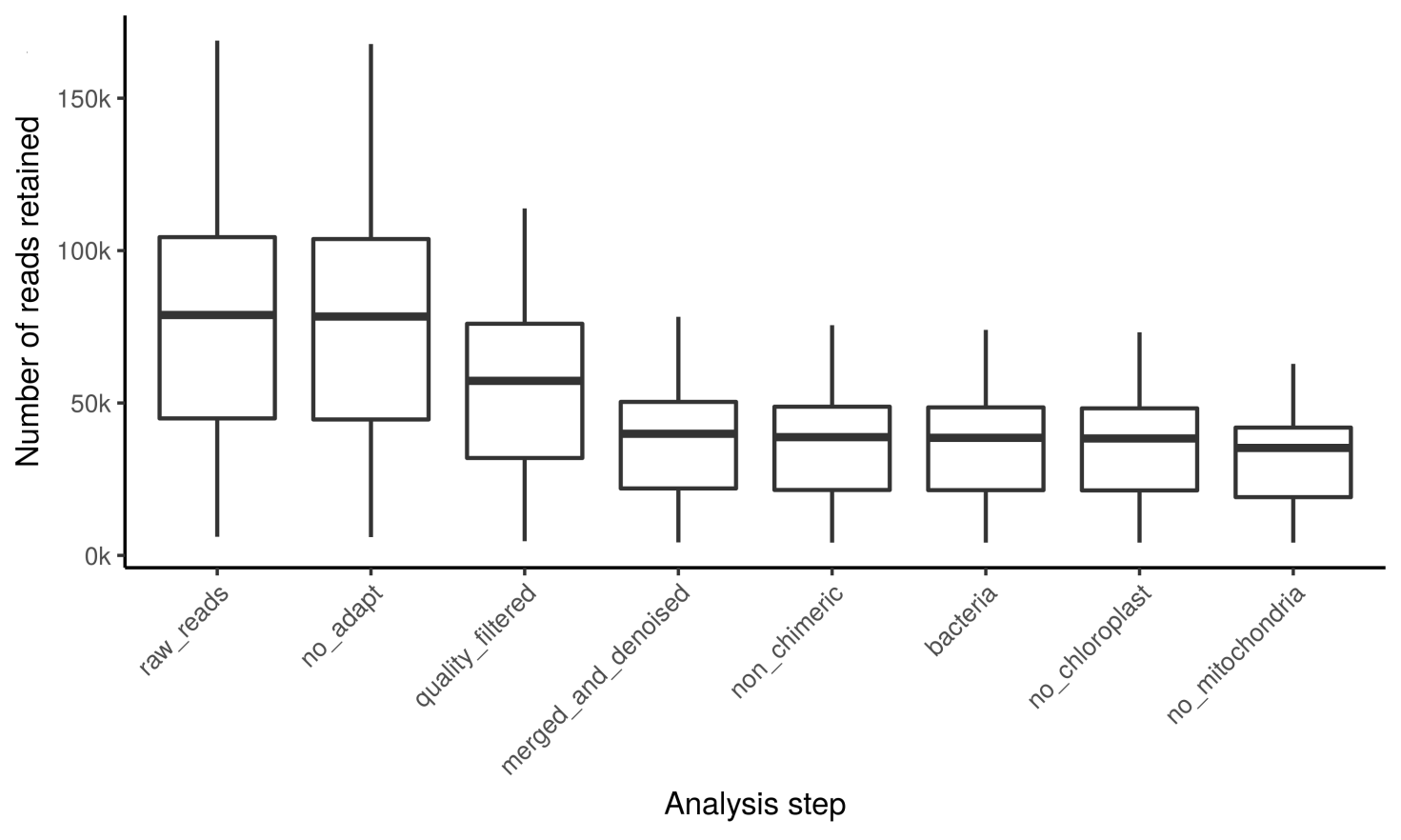


**Figure S2.** **Number of sequences retained at each step of analysis.**

**
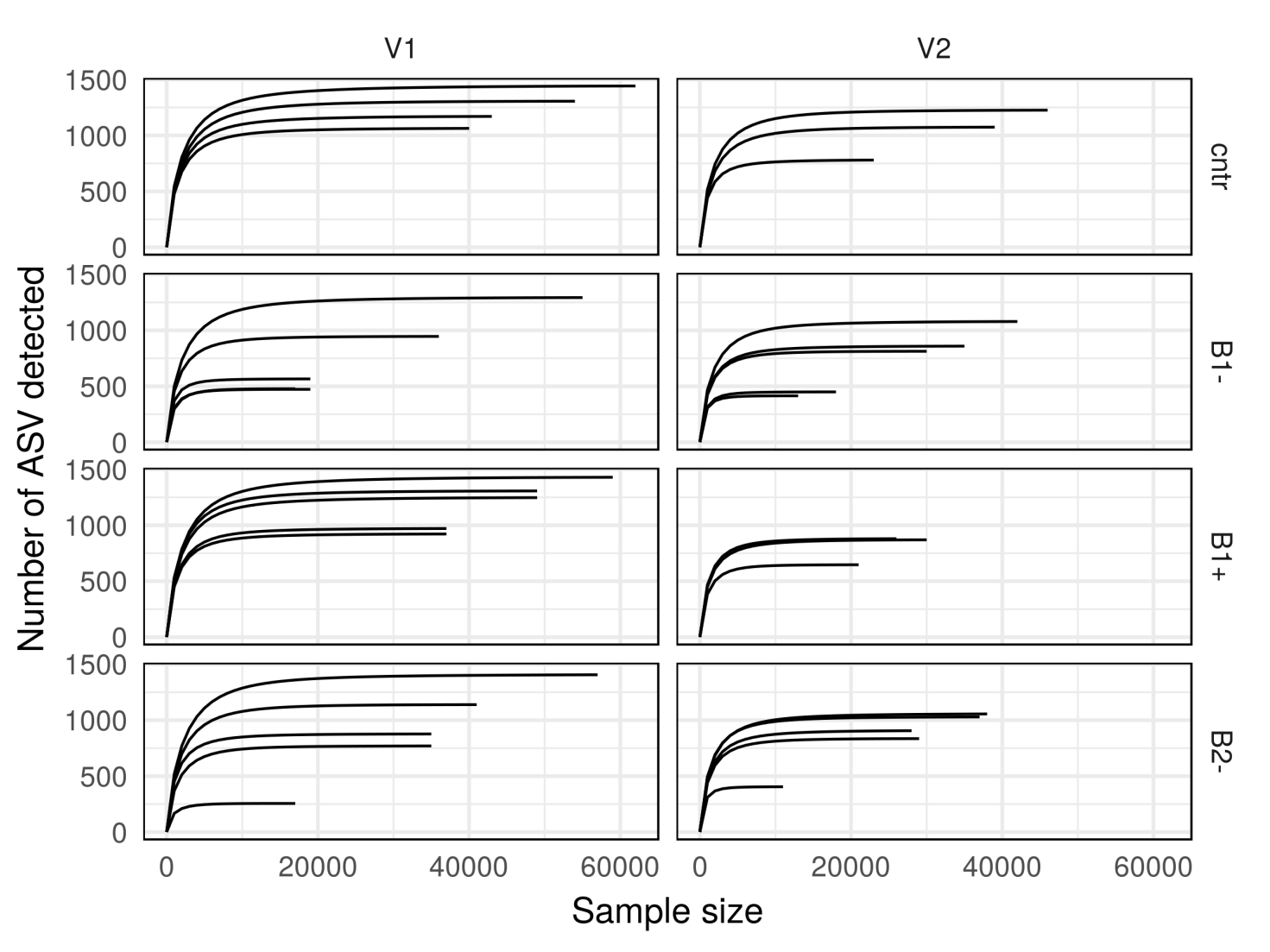
**

**Figure S3.** **Rarefaction analysis.**
