# Supplementary material for: The Impact of Soil-Applied Biochars From Different Vegetal Feedstocks on Durum Wheat Plant Performance and Rhizospheric Bacterial Microbiota in Low Metal-Contaminated Soil
Source: Front Microbiol. 2019 Dec 10;10:2694. doi: 10.3389/fmicb.2019.02694 (PMC6916200; doi:10.3389/fmicb.2019.02694)
Supplement: Supplementary file 1 [file Data_Sheet_1.zip › Supplementary_Material_6_Latini_et_al.docx]

Supplementary Material 6

# Processing of Illumina 16S rDNA sequences

The DNA of one sample was amplified with three different barcodes. The obtained amplicons were sequenced as if they were different samples, to obtain three technical replicates. These samples were co-clustered with all other samples using DADA2 (Callahan et al. 2016) and ASVs were detected accordingly.


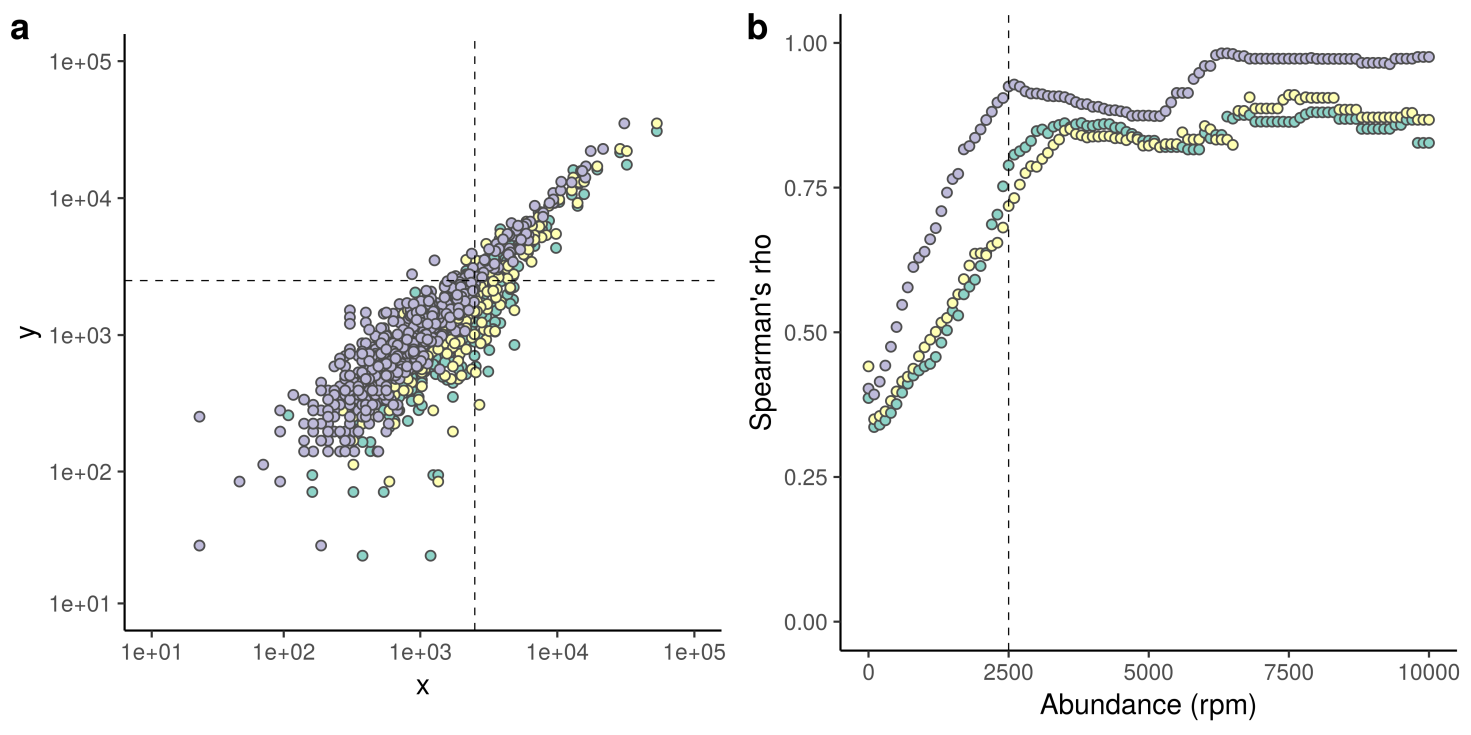
 **Figure S4.** **ASV processing. a.** The count of individual ASVs was normalized within each replicate with the total number of counts per replicate followed multiplied by one million (reads per million, rpm). The abundance of every ASV is plotted for the three pairwise combinations and reported using different colors. The dashed line indicates the threshold arbitrarily chosen to maximise the correlation coefficient reported in b. Each ASV with an abundance value below the given threshold in both samples considered was reported with a lower alpha. **b.** Non-parametric Spearman rank correlation (Spearman’s rho) of data shown in a.

Variants were filtered using a threshold of 2500 rpm. All ASVs with an abundance value higher than the threshold in at least one sample were marked as consistent ASVs, whereas those with all abundance values lower than the threshold were flagged as not-consistent.

Callahan BJ, McMurdie PJ, Rosen MJ, Han AW, Johnson AJA, Holmes SP (2016) DADA2: high-resolution sample inference from Illumina amplicon data. Nat Methods 13: 581. https://dx.doi*.*org*/*10.1038*/*nmeth*.*3869
