# Supplementary material for: The Impact of Soil-Applied Biochars From Different Vegetal Feedstocks on Durum Wheat Plant Performance and Rhizospheric Bacterial Microbiota in Low Metal-Contaminated Soil
Source: Front Microbiol. 2019 Dec 10;10:2694. doi: 10.3389/fmicb.2019.02694 (PMC6916200; doi:10.3389/fmicb.2019.02694)
Supplement: Supplementary file 1 [file Data_Sheet_1.zip › Supplementary_Material_7_Latini_et_al.pdf]

frontiers

Supplementary Material 7

Plant aboveground and belowground fresh and dry weights. Statistical analisys by SPSS.

Total aboveground fresh weight (tAG-FW)

Tests of Normality

	Treatment	Kolmogorov-Smirnov[a]			Shapiro-Wilk		
		Statistic	df	Sig.	Statistic	df	Sig.
Total aboveground FW (g)	V1B1-	,164	9	,200*	,943	9	,609
	V1B1+	,176	9	,200*	,940	9	,580
	V1B2-	,209	9	,200*	,887	9	,185
	V1C	,171	9	,200*	,970	9	,899
	V2B1-	,229	9	,190	,867	9	,115
	V2B1+	,235	9	,162	,873	9	,134
	V2B2-	,126	9	,200*	,943	9	,614
	V2C	,253	9	,100	,850	9	,074

*. This is a lower bound of the true significance.

a. Lilliefors Significance Correction

Oneway

Test of Homogeneity of Variances

Total aboveground FW (g)

Levene Statistic	df1	df2	Sig.
,691	7	64	,679

ANOVA

Total aboveground FW (g)

	Sum of Squares	df	Mean Square	F	Sig.
Between Groups	34,211	7	4,887	15,386	,000
Within Groups	20,329	64	,318		
Total	54,540	71			

Post Hoc Tests

Multiple Comparisons

Dependent Variable: Total aboveground FW (g)

Tukey HSD

(I) Group	(J) Group	Mean Difference (I-J)	Std. Error	Sig.	95% Confidence Interval Lower Bound	Upper Bound
V1C	V1B1(-)	,596111	,265680	,341	-,23636	1,42858
	V1B1(+)	,306444	,265680	,942	-,52603	1,13892
	V1B2(-)	-,939889*	,265680	,016	-1,77236	-,10742
	V2C	,925667*	,265680	,019	,09320	1,75814
	V2B1(-)	,625852	,265680	,281	-,20662	1,45832
	V2B1(+)	,113400	,265680	1,000	-,71907	,94587
	V2B2(-)	-1,113639*	,265680	,002	-1,94611	-,28117
V1B1(-)	V1C	-,596111	,265680	,341	-1,42858	,23636
	V1B1(+)	-,289667	,265680	,957	-1,12214	,54280
	V1B2(-)	-1,536000*	,265680	,000	-2,36847	-,70353
	V2C	,329556	,265680	,916	-,50292	1,16203
	V2B1(-)	,029741	,265680	1,000	-,80273	,86221
	V2B1(+)	-,482711	,265680	,611	-1,31518	,34976
	V2B2(-)	-1,709750*	,265680	,000	-2,54222	-,87728
V1B1(+)	V1C	-,306444	,265680	,942	-1,13892	,52603
	V1B1(-)	,289667	,265680	,957	-,54280	1,12214
	V1B2(-)	-1,246333*	,265680	,000	-2,07880	-,41386
	V2C	,619222	,265680	,294	-,21325	1,45169
	V2B1(-)	,319407	,265680	,928	-,51306	1,15188
	V2B1(+)	-,193044	,265680	,996	-1,02552	,63943
	V2B2(-)	-1,420083*	,265680	,000	-2,25255	-,58761
V1B2(-)	V1C	,939889*	,265680	,016	,10742	1,77236
	V1B1(-)	1,536000*	,265680	,000	,70353	2,36847
	V1B1(+)	1,246333*	,265680	,000	,41386	2,07880
	V2C	1,865556*	,265680	,000	1,03308	2,69803
	V2B1(-)	1,565741*	,265680	,000	,73327	2,39821
	V2B1(+)	1,053289*	,265680	,004	,22082	1,88576
	V2B2(-)	-,173750	,265680	,998	-1,00622	,65872
V2C	V1C	-,925667*	,265680	,019	-1,75814	-,09320
	V1B1(-)	-,329556	,265680	,916	-1,16203	,50292
	V1B1(+)	-,619222	,265680	,294	-1,45169	,21325
	V1B2(-)	-1,865556*	,265680	,000	-2,69803	-1,03308
	V2B1(-)	-,299815	,265680	,948	-1,13229	,53266
	V2B1(+)	-,812267	,265680	,061	-1,64474	,02020
	V2B2(-)	-2,039306*	,265680	,000	-2,87178	-1,20683
V2B1(-)	V1C	-,625852	,265680	,281	-1,45832	,20662
	V1B1(-)	-,029741	,265680	1,000	-,86221	,80273

Multiple Comparisons

Dependent Variable: Total aboveground FW (g)

Tukey HSD

(I) Group	(J) Group	Mean Difference (I-J)	Std. Error	Sig.	95% Confidence Interval	
					Lower Bound	Upper Bound
	V1B1(+)	-,319407	,265680	,928	-1,15188	,51306
	V1B2(-)	-1,565741*	,265680	,000	-2,39821	-,73327
	V2C	,299815	,265680	,948	-,53266	1,13229
	V2B1(+)	-,512452	,265680	,537	-1,34492	,32002
	V2B2(-)	-1,739491*	,265680	,000	-2,57196	-,90702
V2B1(+)	V1C	-,113400	,265680	1,000	-,94587	,71907
	V1B1(-)	,482711	,265680	,611	-,34976	1,31518
	V1B1(+)	,193044	,265680	,996	-,63943	1,02552
	V1B2(-)	-1,053289*	,265680	,004	-1,88576	-,22082
	V2C	,812267	,265680	,061	-,02020	1,64474
	V2B1(-)	,512452	,265680	,537	-,32002	1,34492
	V2B2(-)	-1,227039*	,265680	,000	-2,05951	-,39457
V2B2(-)	V1C	1,113639*	,265680	,002	,28117	1,94611
	V1B1(-)	1,709750*	,265680	,000	,87728	2,54222
	V1B1(+)	1,420083*	,265680	,000	,58761	2,25255
	V1B2(-)	,173750	,265680	,998	-,65872	1,00622
	V2C	2,039306*	,265680	,000	1,20683	2,87178
	V2B1(-)	1,739491*	,265680	,000	,90702	2,57196
	V2B1(+)	1,227039*	,265680	,000	,39457	2,05951

*. The mean difference is significant at the 0.05 level.

Homogeneous Subsets

Total aboveground FW (g)

Tukey HSD[a]

Group	N	Subset for alpha = 0.05		
		1	2	3
V2C	9	2,54244		
V2B1(-)	9	2,84226	2,84226	
V1B1(-)	9	2,87200	2,87200	
V1B1(+)	9	3,16167	3,16167	
V2B1(+)	9	3,35471	3,35471	
V1C	9		3,46811	
V1B2(-)	9			4,40800
V2B2(-)	9			4,58175
Sig.		,061	,281	,998

Means for groups in homogeneous subsets are displayed.

a. Uses Harmonic Mean Sample Size = 9,000.

Comments:
- Data are normally distributed (Shapiro-Wilk Sig. > 0.05)
- There are some outliers but not extreme ones (thus there was no outlier removal)
- Variances are homogeneous (Levene Sig. > 0.05).

ANOVA results:
- Yes, there is at list one significant difference among groups (Sig. = 0.000)
- ($F_{7,64}$ = 15,386; $p < 0.001$).

Tukey multiple comparison results:
- tAG-FW of V1B2- and V2B2- samples differ significantly from other treatments
- There is a significant difference between tAG-FW in V1C and V2C.

Total belowground fresh weight (tBG-FW)

Tests of Normality

	Treatment	Kolmogorov-Smirnov[a]			Shapiro-Wilk		
		Statistic	df	Sig.	Statistic	df	Sig.
Total belowground FW (g)	V1B1-	,137	9	,200*	,978	9	,952
	V1B1+	,244	9	,131	,891	9	,204
	V1B2-	,294	9	,024	,884	9	,172
	V1C	,152	9	,200*	,961	9	,807
	V2B1-	,222	9	,200*	,955	9	,747
	V2B1+	,128	9	,200*	,970	9	,893
	V2B2-	,230	9	,188	,887	9	,185
	V2C	,147	9	,200*	,963	9	,825

*. This is a lower bound of the true significance.

a. Lilliefors Significance Correction

Oneway

Test of Homogeneity of Variances

Total belowground FW (g)

Levene Statistic	df1	df2	Sig.
1,817	7	64	,099

ANOVA

Total belowground FW (g)

	Sum of Squares	df	Mean Square	F	Sig.
Between Groups	,758	7	,108	11,916	,000
Within Groups	,582	64	,009		
Total	1,340	71			

Post Hoc Tests

Multiple Comparisons

Dependent Variable: Total belowground FW (g)

Tukey HSD

(I) Group	(J) Group	Mean Difference (I-J)	Std. Error	Sig.	95% Confidence Interval	
					Lower Bound	Upper Bound
V1C	V1B1(-)	-,138222	,044938	,058	-,27903	,00259
	V1B1(+)	-,120389	,044938	,148	-,26120	,02042
	V1B2(-)	-,246333*	,044938	,000	-,38714	-,10553
	V2C	-,135667	,044938	,067	-,27647	,00514
	V2B1(-)	-,040926	,044938	,984	-,18173	,09988
	V2B1(+)	-,272733*	,044938	,000	-,41354	-,13193
	V2B2(-)	-,307278*	,044938	,000	-,44809	-,16647
V1B1(-)	V1C	,138222	,044938	,058	-,00259	,27903
	V1B1(+)	,017833	,044938	1,000	-,12297	,15864
	V1B2(-)	-,108111	,044938	,256	-,24892	,03270
	V2C	,002556	,044938	1,000	-,13825	,14336
	V2B1(-)	,097296	,044938	,386	-,04351	,23810
	V2B1(+)	-,134511	,044938	,071	-,27532	,00630
	V2B2(-)	-,169056*	,044938	,008	-,30986	-,02825
V1B1(+)	V1C	,120389	,044938	,148	-,02042	,26120
	V1B1(-)	-,017833	,044938	1,000	-,15864	,12297
	V1B2(-)	-,125944	,044938	,112	-,26675	,01486
	V2C	-,015278	,044938	1,000	-,15609	,12553
	V2B1(-)	,079463	,044938	,643	-,06134	,22027
	V2B1(+)	-,152344*	,044938	,025	-,29315	-,01154
	V2B2(-)	-,186889*	,044938	,002	-,32770	-,04608
V1B2(-)	V1C	,246333*	,044938	,000	,10553	,38714
	V1B1(-)	,108111	,044938	,256	-,03270	,24892
	V1B1(+)	,125944	,044938	,112	-,01486	,26675
	V2C	,110667	,044938	,230	-,03014	,25147
	V2B1(-)	,205407*	,044938	,001	,06460	,34622
	V2B1(+)	-,026400	,044938	,999	-,16721	,11441
	V2B2(-)	-,060944	,044938	,873	-,20175	,07986
V2C	V1C	,135667	,044938	,067	-,00514	,27647
	V1B1(-)	-,002556	,044938	1,000	-,14336	,13825
	V1B1(+)	,015278	,044938	1,000	-,12553	,15609

Multiple Comparisons

Dependent Variable: Total belowground FW (g)

Tukey HSD

(I) Group	(J) Group	Mean Difference (I-J)	Std. Error	Sig.	95% Confidence Interval	
					Lower Bound	Upper Bound
	V1B2(-)	-,110667	,044938	,230	-,25147	,03014
	V2B1(-)	,094741	,044938	,421	-,04607	,23555
	V2B1(+)	-,137067	,044938	,062	-,27787	,00374
	V2B2(-)	-,171611*	,044938	,007	-,31242	-,03080
V2B1(-)	V1C	,040926	,044938	,984	-,09988	,18173
	V1B1(-)	-,097296	,044938	,386	-,23810	,04351
	V1B1(+)	-,079463	,044938	,643	-,22027	,06134
	V1B2(-)	-,205407*	,044938	,001	-,34622	-,06460
	V2C	-,094741	,044938	,421	-,23555	,04607
	V2B1(+)	-,231807*	,044938	,000	-,37262	-,09100
	V2B2(-)	-,266352*	,044938	,000	-,40716	-,12554
V2B1(+)	V1C	,272733*	,044938	,000	,13193	,41354
	V1B1(-)	,134511	,044938	,071	-,00630	,27532
	V1B1(+)	,152344*	,044938	,025	,01154	,29315
	V1B2(-)	,026400	,044938	,999	-,11441	,16721
	V2C	,137067	,044938	,062	-,00374	,27787
	V2B1(-)	,231807*	,044938	,000	,09100	,37262
	V2B2(-)	-,034544	,044938	,994	-,17535	,10626
V2B2(-)	V1C	,307278*	,044938	,000	,16647	,44809
	V1B1(-)	,169056*	,044938	,008	,02825	,30986
	V1B1(+)	,186889*	,044938	,002	,04608	,32770
	V1B2(-)	,060944	,044938	,873	-,07986	,20175
	V2C	,171611*	,044938	,007	,03080	,31242
	V2B1(-)	,266352*	,044938	,000	,12554	,40716
	V2B1(+)	,034544	,044938	,994	-,10626	,17535

*. The mean difference is significant at the 0.05 level.

Homogeneous Subsets

Total belowground FW (g)

Tukey HSD[a]

Group	N	Subset for alpha = 0.05			
		1	2	3	4
V1C	9	,28100			
V2B1(-)	9	,32193			
V1B1(+)	9	,40139	,40139		
V2C	9	,41667	,41667	,41667	
V1B1(-)	9	,41922	,41922	,41922	
V1B2(-)	9		,52733	,52733	,52733
V2B1(+)	9			,55373	,55373
V2B2(-)	9				,58828
Sig.		,058	,112	,062	,873

Means for groups in homogeneous subsets are displayed.

a. Uses Harmonic Mean Sample Size = 9,000.

Comments:
- Data are normally distributed (Shapiro-Wilk Sig. > 0.05)
- There are some outliers and also some extreme ones
- Finally V1B2- (P4) was removed as outlier and replaced with the average of the remaining eig ht plants
- Variances are homogeneous (Levene Sig. > 0.05).

ANOVA results:
- Yes, there is at list one significant difference among groups (Sig. = 0.000)
- ($F_{7,64}$ = 11,966; $p < 0.001$).

Tukey multiple comparison results:
- V1C is different from V1B2-, so B2- has an effect on tBG-FW of V1
- V2C is different from V2B2-, so B2- has an effect on tBG-FW of V2
- B1+ and B1- have a different effect on V2, but this is not true for V1.

Total aboveground dry weight (tAG-DW)

Tests of Normality

	Treatment	Kolmogorov-Smirnov[a]			Shapiro-Wilk		
		Statistic	df	Sig.	Statistic	df	Sig.
Total aboveground DW (g)	V1B1-	,183	9	,200*	,931	9	,489
	V1B1+	,205	9	,200*	,958	9	,780
	V1B2-	,253	9	,100	,849	9	,073
	V1C	,160	9	,200*	,932	9	,504
	V2B1-	,239	9	,146	,844	9	,064
	V2B1+	,203	9	,200*	,947	9	,662
	V2B2-	,148	9	,200*	,952	9	,711
	V2C	,159	9	,200*	,953	9	,723

*. This is a lower bound of the true significance.

a. Lilliefors Significance Correction

Oneway

Test of Homogeneity of Variances

Total aboveground DW (g)

Levene Statistic	df1	df2	Sig.
1,677	7	64	,131

Robust Tests of Equality of Means

Total aboveground DW (g)

	Statistic[a]	df1	df2	Sig.
Welch	32,648	7	27,198	,000

a. Asymptotically F distributed.

ANOVA

Total aboveground DW (g)

	Sum of Squares	df	Mean Square	F	Sig.
Between Groups	,661	7	,094	34,118	,000
Within Groups	,177	64	,003		
Total	,838	71			

Multiple Comparisons

Dependent Variable: Total aboveground DW (g)

Games-Howell

(I) Group	(J) Group	Mean Difference (I-J)	Std. Error	Sig.	95% Confidence Interval Lower Bound	95% Confidence Interval Upper Bound
V1C	V1B1(-)	,070000	,027534	,275	-,03256	,17256
	V1B1(+)	,039333	,027756	,832	-,06350	,14216
	V1B2(-)	-,094903	,031715	,123	-,20613	,01633
	V2C	,053556	,032205	,709	-,05905	,16616
	V2B1(-)	,042593	,029070	,811	-,06237	,14755
	V2B1(+)	-,017911	,031523	,999	-,12862	,09280
	V2B2(-)	-,235736*	,031766	,000	-,34711	-,12436
V1B1(-)	V1C	-,070000	,027534	,275	-,17256	,03256
	V1B1(+)	-,030667	,014460	,443	-,08075	,01942
	V1B2(-)	-,164903*	,021083	,000	-,24098	-,08883
	V2C	-,016444	,021814	,993	-,09551	,06262
	V2B1(-)	-,027407	,016845	,729	-,08646	,03165
	V2B1(+)	-,087911*	,020793	,018	-,16281	-,01302
	V2B2(-)	-,305736*	,021161	,000	-,38213	-,22934
V1B1(+)	V1C	-,039333	,027756	,832	-,14216	,06350
	V1B1(-)	,030667	,014460	,443	-,01942	,08075
	V1B2(-)	-,134236*	,021372	,001	-,21090	-,05758
	V2C	,014222	,022094	,997	-,06538	,09382
	V2B1(-)	,003259	,017205	1,000	-,05682	,06333
	V2B1(+)	-,057244	,021086	,202	-,13274	,01825
	V2B2(-)	-,275069*	,021449	,000	-,35204	-,19810
V1B2(-)	V1C	,094903	,031715	,123	-,01633	,20613
	V1B1(-)	,164903*	,021083	,000	,08883	,24098
	V1B1(+)	,134236*	,021372	,001	,05758	,21090
	V2C	,148458*	,026899	,001	,05531	,24161
	V2B1(-)	,137495*	,023053	,001	,05670	,21829
	V2B1(+)	,076992	,026078	,126	-,01330	,16728
	V2B2(-)	-,140833*	,026371	,001	-,23214	-,04953
V2C	V1C	-,053556	,032205	,709	-,16616	,05905
	V1B1(-)	,016444	,021814	,993	-,06262	,09551
	V1B1(+)	-,014222	,022094	,997	-,09382	,06538
	V1B2(-)	-,148458*	,026899	,001	-,24161	-,05531
	V2B1(-)	-,010963	,023723	1,000	-,09438	,07246
	V2B1(+)	-,071467	,026672	,199	-,16386	,02092
	V2B2(-)	-,289292*	,026959	,000	-,38265	-,19594
V2B1(-)	V1C	-,042593	,029070	,811	-,14755	,06237
	V1B1(-)	,027407	,016845	,729	-,03165	,08646

Multiple Comparisons

Dependent Variable: Total aboveground DW (g)

Games-Howell

(I) Group	(J) Group	Mean Difference (I-J)	Std. Error	Sig.	95% Confidence Interval Lower Bound	95% Confidence Interval Upper Bound
	V1B1(+)	-,003259	,017205	1,000	-,06333	,05682
	V1B2(-)	-,137495*	,023053	,001	-,21829	-,05670
	V2C	,010963	,023723	1,000	-,07246	,09438
	V2B1(+)	-,060504	,022788	,211	-,14027	,01926
	V2B2(-)	-,278329*	,023124	,000	-,35940	-,19726
V2B1(+)	V1C	,017911	,031523	,999	-,09280	,12862
	V1B1(-)	,087911*	,020793	,018	,01302	,16281
	V1B1(+)	,057244	,021086	,202	-,01825	,13274
	V1B2(-)	-,076992	,026078	,126	-,16728	,01330
	V2C	,071467	,026672	,199	-,02092	,16386
	V2B1(-)	,060504	,022788	,211	-,01926	,14027
	V2B2(-)	-,217825*	,026140	,000	-,30833	-,12732
V2B2(-)	V1C	,235736*	,031766	,000	,12436	,34711
	V1B1(-)	,305736*	,021161	,000	,22934	,38213
	V1B1(+)	,275069*	,021449	,000	,19810	,35204
	V1B2(-)	,140833*	,026371	,001	,04953	,23214
	V2C	,289292*	,026959	,000	,19594	,38265
	V2B1(-)	,278329*	,023124	,000	,19726	,35940
	V2B1(+)	,217825*	,026140	,000	,12732	,30833

*. The mean difference is significant at the 0.05 level.

Post Hoc Tests

Multiple Comparisons

Dependent Variable: Total aboveground DW (g)

Tukey HSD

(I) Group	(J) Group	Mean Difference (I-J)	Std. Error	Sig.	95% Confidence Interval	
					Lower Bound	Upper Bound
V1C	V1B1(-)	,070000	,024804	,108	-,00772	,14772
	V1B1(+)	,039333	,024804	,757	-,03839	,11705
	V1B2(-)	-,094903*	,024804	,007	-,17262	-,01718
	V2C	,053556	,024804	,390	-,02417	,13128
	V2B1(-)	,042593	,024804	,676	-,03513	,12031
	V2B1(+)	-,017911	,024804	,996	-,09563	,05981
	V2B2(-)	-,235736*	,024804	,000	-,31346	-,15802
V1B1(-)	V1C	-,070000	,024804	,108	-,14772	,00772
	V1B1(+)	-,030667	,024804	,918	-,10839	,04705
	V1B2(-)	-,164903*	,024804	,000	-,24262	-,08718
	V2C	-,016444	,024804	,998	-,09417	,06128
	V2B1(-)	-,027407	,024804	,953	-,10513	,05031
	V2B1(+)	-,087911*	,024804	,016	-,16563	-,01019
	V2B2(-)	-,305736*	,024804	,000	-,38346	-,22802
V1B1(+)	V1C	-,039333	,024804	,757	-,11705	,03839
	V1B1(-)	,030667	,024804	,918	-,04705	,10839
	V1B2(-)	-,134236*	,024804	,000	-,21196	-,05652
	V2C	,014222	,024804	,999	-,06350	,09194
	V2B1(-)	,003259	,024804	1,000	-,07446	,08098
	V2B1(+)	-,057244	,024804	,306	-,13497	,02048
	V2B2(-)	-,275069*	,024804	,000	-,35279	-,19735
V1B2(-)	V1C	,094903*	,024804	,007	,01718	,17262
	V1B1(-)	,164903*	,024804	,000	,08718	,24262
	V1B1(+)	,134236*	,024804	,000	,05652	,21196
	V2C	,148458*	,024804	,000	,07074	,22618
	V2B1(-)	,137495*	,024804	,000	,05977	,21522
	V2B1(+)	,076992	,024804	,054	-,00073	,15471
	V2B2(-)	-,140833*	,024804	,000	-,21855	-,06311
V2C	V1C	-,053556	,024804	,390	-,13128	,02417
	V1B1(-)	,016444	,024804	,998	-,06128	,09417
	V1B1(+)	-,014222	,024804	,999	-,09194	,06350
	V1B2(-)	-,148458*	,024804	,000	-,22618	-,07074
	V2B1(-)	-,010963	,024804	1,000	-,08868	,06676
	V2B1(+)	-,071467	,024804	,094	-,14919	,00625
	V2B2(-)	-,289292*	,024804	,000	-,36701	-,21157
V2B1(-)	V1C	-,042593	,024804	,676	-,12031	,03513
	V1B1(-)	,027407	,024804	,953	-,05031	,10513

Multiple Comparisons

Dependent Variable: Total aboveground DW (g)

Tukey HSD

(I) Group	(J) Group	Mean Difference (I-J)	Std. Error	Sig.	95% Confidence Interval	
					Lower Bound	Upper Bound
	V1B1(+)	-,003259	,024804	1,000	-,08098	,07446
	V1B2(-)	-,137495*	,024804	,000	-,21522	-,05977
	V2C	,010963	,024804	1,000	-,06676	,08868
	V2B1(+)	-,060504	,024804	,241	-,13822	,01722
	V2B2(-)	-,278329*	,024804	,000	-,35605	-,20061
V2B1(+)	V1C	,017911	,024804	,996	-,05981	,09563
	V1B1(-)	,087911*	,024804	,016	,01019	,16563
	V1B1(+)	,057244	,024804	,306	-,02048	,13497
	V1B2(-)	-,076992	,024804	,054	-,15471	,00073
	V2C	,071467	,024804	,094	-,00625	,14919
	V2B1(-)	,060504	,024804	,241	-,01722	,13822
	V2B2(-)	-,217825*	,024804	,000	-,29555	-,14010
V2B2(-)	V1C	,235736*	,024804	,000	,15802	,31346
	V1B1(-)	,305736*	,024804	,000	,22802	,38346
	V1B1(+)	,275069*	,024804	,000	,19735	,35279
	V1B2(-)	,140833*	,024804	,000	,06311	,21855
	V2C	,289292*	,024804	,000	,21157	,36701
	V2B1(-)	,278329*	,024804	,000	,20061	,35605
	V2B1(+)	,217825*	,024804	,000	,14010	,29555

*. The mean difference is significant at the 0.05 level.

Homogeneous Subsets

Total aboveground DW (g)

Tukey HSD[a]

Group	N	Subset for alpha = 0.05			
		1	2	3	4
V1B1(-)	9	,38022			
V2C	9	,39667	,39667		
V2B1(-)	9	,40763	,40763		
V1B1(+)	9	,41089	,41089		
V1C	9	,45022	,45022		
V2B1(+)	9		,46813	,46813	
V1B2(-)	9			,54513	
V2B2(-)	9				,68596
Sig.		,108	,094	,054	1,000

Means for groups in homogeneous subsets are displayed.

a. Uses Harmonic Mean Sample Size = 9,000.

Comments:
- Data are normally distributed (Shapiro-Wilk Sig. > 0.05)
- There are some outliers but no extreme ones (thus there was no outlier removal)
- Variances are NOT homogeneous (Levene Sig. = 0.131), so Welch ANOVA has been perform ed followed by Games-Howell as post-hoc test.

ANOVA results:
- Yes, there is at list one significant difference among groups (Sig. = 0.000)
- ($F_{7,64}$ = 34,118; $p < 0.001$).

Games-Howell multiple comparison results:
- The results of post-hoc analysis with Games-Howell are the same of reported Tukey test, with a single exception (with Tukey V1C differs from V1B2-)
- V2B2- is different from all other samples
- B2- has an effect on both V1 and V2 with Tukey test, but only on V2 with Games-Howell test.
- B1- and B2- have a different effect on V1 and V2.

Total belowground dry weight (tBG-DW)

Tests of Normality

	Treatment	Kolmogorov-Smirnov[a]			Shapiro-Wilk		
		Statistic	df	Sig.	Statistic	df	Sig.
Total belowground DW (g)	V1B1-	,124	9	,200*	,977	9	,948
	V1B1+	,164	9	,200*	,901	9	,257
	V1B2-	,281	9	,039	,875	9	,139
	V1C	,222	9	,200*	,909	9	,309
	V2B1-	,214	9	,200*	,865	9	,109
	V2B1+	,165	9	,200*	,896	9	,230
	V2B2-	,245	9	,128	,865	9	,108
	V2C	,174	9	,200*	,963	9	,831

*. This is a lower bound of the true significance.

a. Lilliefors Significance Correction

Oneway

Test of Homogeneity of Variances

Total belowground DW (g)

Levene Statistic	df1	df2	Sig.
,863	7	64	,541

Robust Tests of Equality of Means

Total belowground DW (g)

	Statistic[a]	df1	df2	Sig.
Welch	5,019	7	27,369	,001

a. Asymptotically F distributed.

ANOVA

Total belowground DW (g)

	Sum of Squares	df	Mean Square	F	Sig.
Between Groups	,068	7	,010	6,131	,000
Within Groups	,102	64	,002		
Total	,170	71			

Total belowground dry weight (tBG-DW)

Multiple Comparisons

Dependent Variable: Total belowground DW (g)

Games-Howell

(I) Group	(J) Group	Mean Difference (I-J)	Std. Error	Sig.	95% Confidence Interval Lower Bound	95% Confidence Interval Upper Bound
V1C	V1B1(-)	,008000	,019870	1,000	-,06088	,07688
	V1B1(+)	,021556	,018591	,932	-,04337	,08648
	V1B2(-)	,024111	,017920	,867	-,03895	,08717
	V2C	,010000	,021320	1,000	-,06384	,08384
	V2B1(-)	,034556	,018142	,568	-,02910	,09821
	V2B1(+)	-,017333	,020616	,988	-,08871	,05405
	V2B2(-)	-,070000	,021086	,065	-,14301	,00301
V1B1(-)	V1C	-,008000	,019870	1,000	-,07688	,06088
	V1B1(+)	,013556	,017523	,992	-,04732	,07443
	V1B2(-)	,016111	,016809	,974	-,04261	,07484
	V2C	,002000	,020395	1,000	-,06881	,07281
	V2B1(-)	,026556	,017046	,767	-,03286	,08597
	V2B1(+)	-,025333	,019658	,890	-,09345	,04278
	V2B2(-)	-,078000*	,020151	,023	-,14791	-,00809
V1B1(+)	V1C	-,021556	,018591	,932	-,08648	,04337
	V1B1(-)	-,013556	,017523	,992	-,07443	,04732
	V1B2(-)	,002556	,015277	1,000	-,05041	,05552
	V2C	-,011556	,019152	,998	-,07865	,05553
	V2B1(-)	,013000	,015536	,988	-,04082	,06682
	V2B1(+)	-,038889	,018365	,446	-,10294	,02516
	V2B2(-)	-,091556*	,018891	,004	-,15763	-,02548
V1B2(-)	V1C	-,024111	,017920	,867	-,08717	,03895
	V1B1(-)	-,016111	,016809	,974	-,07484	,04261
	V1B1(+)	-,002556	,015277	1,000	-,05552	,05041
	V2C	-,014111	,018501	,993	-,07948	,05125
	V2B1(-)	,010444	,014727	,995	-,04055	,06144
	V2B1(+)	-,041444	,017685	,335	-,10358	,02069
	V2B2(-)	-,094111*	,018231	,003	-,15840	-,02982
V2C	V1C	-,010000	,021320	1,000	-,08384	,06384
	V1B1(-)	-,002000	,020395	1,000	-,07281	,06881
	V1B1(+)	,011556	,019152	,998	-,05553	,07865
	V1B2(-)	,014111	,018501	,993	-,05125	,07948
	V2B1(-)	,024556	,018716	,880	-,04136	,09047
	V2B1(+)	-,027333	,021123	,888	-,10051	,04584
	V2B2(-)	-,080000*	,021583	,031	-,15473	-,00527
V2B1(-)	V1C	-,034556	,018142	,568	-,09821	,02910
	V1B1(-)	-,026556	,017046	,767	-,08597	,03286
	V1B1(+)	-,013000	,015536	,988	-,06682	,04082
	V1B2(-)	-,010444	,014727	,995	-,06144	,04055

Total belowground dry weight (tBG-DW)

Multiple Comparisons

Dependent Variable: Total belowground DW (g)

Games-Howell

(I) Group	(J) Group	Mean Difference (I-J)	Std. Error	Sig.	95% Confidence Interval	
					Lower Bound	Upper Bound
	V2C	-,024556	,018716	,880	-,09047	,04136
	V2B1(+)	-,051889	,017910	,144	-,11464	,01086
	V2B2(-)	-,104556*	,018449	,001	-,16942	-,03969
V2B1(+)	V1C	,017333	,020616	,988	-,05405	,08871
	V1B1(-)	,025333	,019658	,890	-,04278	,09345
	V1B1(+)	,038889	,018365	,446	-,02516	,10294
	V1B2(-)	,041444	,017685	,335	-,02069	,10358
	V2C	,027333	,021123	,888	-,04584	,10051
	V2B1(-)	,051889	,017910	,144	-,01086	,11464
	V2B2(-)	-,052667	,020887	,254	-,12500	,01967
V2B2(-)	V1C	,070000	,021086	,065	-,00301	,14301
	V1B1(-)	,078000*	,020151	,023	,00809	,14791
	V1B1(+)	,091556*	,018891	,004	,02548	,15763
	V1B2(-)	,094111*	,018231	,003	,02982	,15840
	V2C	,080000*	,021583	,031	,00527	,15473
	V2B1(-)	,104556*	,018449	,001	,03969	,16942
	V2B1(+)	,052667	,020887	,254	-,01967	,12500

*. The mean difference is significant at the 0.05 level.

Total belowground dry weight (tBG-DW)

Multiple Comparisons

Dependent Variable: Total belowground DW (g)

Tukey HSD

(I) Group	(J) Group	Mean Difference (I-J)	Std. Error	Sig.	95% Confidence Interval Lower Bound	95% Confidence Interval Upper Bound
V1C	V1B1(-)	,008000	,018807	1,000	-,05093	,06693
	V1B1(+)	,021556	,018807	,944	-,03737	,08048
	V1B2(-)	,024111	,018807	,902	-,03482	,08304
	V2C	,010000	,018807	,999	-,04893	,06893
	V2B1(-)	,034556	,018807	,598	-,02437	,09348
	V2B1(+)	-,017333	,018807	,983	-,07626	,04159
	V2B2(-)	-,070000*	,018807	,009	-,12893	-,01107
V1B1(-)	V1C	-,008000	,018807	1,000	-,06693	,05093
	V1B1(+)	,013556	,018807	,996	-,04537	,07248
	V1B2(-)	,016111	,018807	,989	-,04282	,07504
	V2C	,002000	,018807	1,000	-,05693	,06093
	V2B1(-)	,026556	,018807	,848	-,03237	,08548
	V2B1(+)	-,025333	,018807	,877	-,08426	,03359
	V2B2(-)	-,078000*	,018807	,002	-,13693	-,01907
V1B1(+)	V1C	-,021556	,018807	,944	-,08048	,03737
	V1B1(-)	-,013556	,018807	,996	-,07248	,04537
	V1B2(-)	,002556	,018807	1,000	-,05637	,06148
	V2C	-,011556	,018807	,999	-,07048	,04737
	V2B1(-)	,013000	,018807	,997	-,04593	,07193
	V2B1(+)	-,038889	,018807	,446	-,09782	,02004
	V2B2(-)	-,091556*	,018807	,000	-,15048	-,03263
V1B2(-)	V1C	-,024111	,018807	,902	-,08304	,03482
	V1B1(-)	-,016111	,018807	,989	-,07504	,04282
	V1B1(+)	-,002556	,018807	1,000	-,06148	,05637
	V2C	-,014111	,018807	,995	-,07304	,04482
	V2B1(-)	,010444	,018807	,999	-,04848	,06937
	V2B1(+)	-,041444	,018807	,363	-,10037	,01748
	V2B2(-)	-,094111*	,018807	,000	-,15304	-,03518
V2C	V1C	-,010000	,018807	,999	-,06893	,04893
	V1B1(-)	-,002000	,018807	1,000	-,06093	,05693
	V1B1(+)	,011556	,018807	,999	-,04737	,07048
	V1B2(-)	,014111	,018807	,995	-,04482	,07304
	V2B1(-)	,024556	,018807	,893	-,03437	,08348
	V2B1(+)	-,027333	,018807	,829	-,08626	,03159
	V2B2(-)	-,080000*	,018807	,002	-,13893	-,02107
V2B1(-)	V1C	-,034556	,018807	,598	-,09348	,02437
	V1B1(-)	-,026556	,018807	,848	-,08548	,03237
	V1B1(+)	-,013000	,018807	,997	-,07193	,04593
	V1B2(-)	-,010444	,018807	,999	-,06937	,04848

Total belowground dry weight (tBG-DW)

Multiple Comparisons

Dependent Variable: Total belowground DW (g)

Tukey HSD

(I) Group	(J) Group	Mean Difference (I-J)	Std. Error	Sig.	95% Confidence Interval	
					Lower Bound	Upper Bound
	V2C	-,024556	,018807	,893	-,08348	,03437
	V2B1(+)	-,051889	,018807	,124	-,11082	,00704
	V2B2(-)	-,104556*	,018807	,000	-,16348	-,04563
V2B1(+)	V1C	,017333	,018807	,983	-,04159	,07626
	V1B1(-)	,025333	,018807	,877	-,03359	,08426
	V1B1(+)	,038889	,018807	,446	-,02004	,09782
	V1B2(-)	,041444	,018807	,363	-,01748	,10037
	V2C	,027333	,018807	,829	-,03159	,08626
	V2B1(-)	,051889	,018807	,124	-,00704	,11082
	V2B2(-)	-,052667	,018807	,113	-,11159	,00626
V2B2(-)	V1C	,070000*	,018807	,009	,01107	,12893
	V1B1(-)	,078000*	,018807	,002	,01907	,13693
	V1B1(+)	,091556*	,018807	,000	,03263	,15048
	V1B2(-)	,094111*	,018807	,000	,03518	,15304
	V2C	,080000*	,018807	,002	,02107	,13893
	V2B1(-)	,104556*	,018807	,000	,04563	,16348
	V2B1(+)	,052667	,018807	,113	-,00626	,11159

*. The mean difference is significant at the 0.05 level.

Homogeneous Subsets

Total belowground DW (g)

Tukey HSD[a]

Group	N	Subset for alpha = 0.05	
		1	2
V2B1(-)	9	,08178	
V1B2(-)	9	,09222	
V1B1(+)	9	,09478	
V2C	9	,10633	
V1B1(-)	9	,10833	
V1C	9	,11633	
V2B1(+)	9	,13367	,13367
V2B2(-)	9		,18633
Sig.		,124	,113

Means for groups in homogeneous subsets are displayed.

a. Uses Harmonic Mean Sample Size = 9,000.

Total belowground dry weight (tBG-DW)

Comments:
- Data are normally distributed (Shapiro-Wilk Sig. > 0.05)
- There are no outliers
- Variances are NOT homogeneous (Levene Sig. = 0.541), so Welch ANOVA has been perform ed followed by Games-Howell as post-hoc test.

ANOVA results:
- Yes, there is at list one significant difference among groups (Sig. = 0.000)
- ($F_{7,64}$ = 6,131; $p < 0.001$).

Games-Howell multiple comparison results:
- The results of post-hoc analysis with Games-Howell are the same of reported Tukey test, with a single exception (with Tukey V1C differs from V2B2-)
- tBG-DW of V2B2- samples differ significantly from all other treatments, with the exception of V2 B1+ and V1C.
- V2B2- doesn't differ significantly from V1C with Games-Howell post-hoc, but it does with Tukey post-hoc.
