# Supplementary material for: The Impact of Soil-Applied Biochars From Different Vegetal Feedstocks on Durum Wheat Plant Performance and Rhizospheric Bacterial Microbiota in Low Metal-Contaminated Soil
Source: Front Microbiol. 2019 Dec 10;10:2694. doi: 10.3389/fmicb.2019.02694 (PMC6916200; doi:10.3389/fmicb.2019.02694)
Supplement: Supplementary file 1 [file Data_Sheet_1.zip › Supplementary_Material_8_Latini_et_al.pdf]

Supplementary Materials 8

Plant morphometric traits. Statistical analisys by SP SS.

Tests of Normality

	Treatment	Kolmogorov-Smirnov[a]			Shapiro-Wilk		
		Statistic	df	Sig.	Statistic	df	Sig.
Plant Height (cm)	V1B1-	,156	9	,200*	,969	9	,885
	V1B1+	,189	9	,200*	,913	9	,338
	V1B2-	,271	9	,056	,869	9	,121
	V1C	,194	9	,200*	,928	9	,467
	V2B1-	,160	9	,200*	,957	9	,771
	V2B1+	,184	9	,200*	,940	9	,577
	V2B2-	,140	9	,200*	,949	9	,681
	V2C	,228	9	,197	,935	9	,527
Flag Leaf Lenght (cm)	V1B1-	,149	9	,200*	,976	9	,940
	V1B1+	,181	9	,200*	,907	9	,294
	V1B2-	,311	9	,012	,828	9	,043
	V1C	,201	9	,200*	,930	9	,481
	V2B1-	,183	9	,200*	,912	9	,329
	V2B1+	,180	9	,200*	,962	9	,816
	V2B2-	,179	9	,200*	,943	9	,616
	V2C	,167	9	,200*	,917	9	,371
maximum Flag Leaf Width (cm)	V1B1-	,335	9	,004	,748	9	,005
	V1B1+	,338	9	,004	,704	9	,002
	V1B2-	,297	9	,021	,813	9	,028
	V1C	,284	9	,035	,863	9	,102
	V2B1-	,297	9	,021	,813	9	,028
	V2B1+	,248	9	,116	,913	9	,338
	V2B2-	,259	9	,083	,844	9	,065
	V2C	,255	9	,096	,940	9	,586

*. This is a lower bound of the true significance.

a. Lilliefors Significance Correction

Oneway

Test of Homogeneity of Variances

	Levene Statistic	df1	df2	Sig.
Plant Height (cm)	,287	7	64	,957
Flag Leaf Lenght (cm)	1,757	7	64	,112

ANOVA

		Sum of Squares	df	Mean Square	F	Sig.
Plant Height (cm)	Between Groups	1326,494	7	189,499	42,466	,000
	Within Groups	285,591	64	4,462		
	Total	1612,086	71			
Flag Leaf Lenght (cm)	Between Groups	244,290	7	34,899	10,799	,000
	Within Groups	206,831	64	3,232		
	Total	451,122	71			

Post Hoc Tests

Multiple Comparisons

Tukey HSD

Dependent Variable	(I) Group	(J) Group	Mean Difference (I-J)	Std. Error	Sig.	95% Confidence Interval	
						Lower Bound	Upper Bound
Plant Height (cm)	V1C	V1B1(-)	-4,0456*	,9958	,003	-7,166	-,925
		V1B1(+)	-2,0867	,9958	,429	-5,207	1,034
		V1B2(-)	-7,2867*	,9958	,000	-10,407	-4,166
		V2C	-6,6500*	,9958	,000	-9,770	-3,530
		V2B1(-)	-10,6000*	,9958	,000	-13,720	-7,480
		V2B1(+)	-6,5622*	,9958	,000	-9,682	-3,442
		V2B2(-)	-14,3867*	,9958	,000	-17,507	-11,266
	V1B1(-)	V1C	4,0456*	,9958	,003	,925	7,166
		V1B1(+)	1,9589	,9958	,512	-1,161	5,079
		V1B2(-)	-3,2411*	,9958	,036	-6,361	-,121
		V2C	-2,6044	,9958	,170	-5,725	,516
		V2B1(-)	-6,5544*	,9958	,000	-9,675	-3,434
		V2B1(+)	-2,5167	,9958	,203	-5,637	,604
		V2B2(-)	-10,3411*	,9958	,000	-13,461	-7,221
	V1B1(+)	V1C	2,0867	,9958	,429	-1,034	5,207
		V1B1(-)	-1,9589	,9958	,512	-5,079	1,161
		V1B2(-)	-5,2000*	,9958	,000	-8,320	-2,080
		V2C	-4,5633*	,9958	,001	-7,684	-1,443
		V2B1(-)	-8,5133*	,9958	,000	-11,634	-5,393
		V2B1(+)	-4,4756*	,9958	,001	-7,596	-1,355
		V2B2(-)	-12,3000*	,9958	,000	-15,420	-9,180
	V1B2(-)	V1C	7,2867*	,9958	,000	4,166	10,407
		V1B1(-)	3,2411*	,9958	,036	,121	6,361
		V1B1(+)	5,2000*	,9958	,000	2,080	8,320
		V2C	,6367	,9958	,998	-2,484	3,757
		V2B1(-)	-3,3133*	,9958	,030	-6,434	-,193
		V2B1(+)	,7244	,9958	,996	-2,396	3,845
		V2B2(-)	-7,1000*	,9958	,000	-10,220	-3,980
	V2C	V1C	6,6500*	,9958	,000	3,530	9,770
		V1B1(-)	2,6044	,9958	,170	-,516	5,725
		V1B1(+)	4,5633*	,9958	,001	1,443	7,684
		V1B2(-)	-,6367	,9958	,998	-3,757	2,484
		V2B1(-)	-3,9500*	,9958	,004	-7,070	-,830
		V2B1(+)	,0878	,9958	1,000	-3,032	3,208
		V2B2(-)	-7,7367*	,9958	,000	-10,857	-4,616

Multiple Comparisons

Tukey HSD

Dependent Variable	(I) Group	(J) Group	Mean Difference (I-J)	Std. Error	Sig.	95% Confidence Interval	
						Lower Bound	Upper Bound
	V2B1(-)	V1C	10,6000*	,9958	,000	7,480	13,720
		V1B1(-)	6,5544*	,9958	,000	3,434	9,675
		V1B1(+)	8,5133*	,9958	,000	5,393	11,634
		V1B2(-)	3,3133*	,9958	,030	,193	6,434
		V2C	3,9500*	,9958	,004	,830	7,070
		V2B1(+)	4,0378*	,9958	,003	,918	7,158
		V2B2(-)	-3,7867*	,9958	,007	-6,907	-,666
	V2B1(+)	V1C	6,5622*	,9958	,000	3,442	9,682
		V1B1(-)	2,5167	,9958	,203	-,604	5,637
		V1B1(+)	4,4756*	,9958	,001	1,355	7,596
		V1B2(-)	-,7244	,9958	,996	-3,845	2,396
		V2C	-,0878	,9958	1,000	-3,208	3,032
		V2B1(-)	-4,0378*	,9958	,003	-7,158	-,918
		V2B2(-)	-7,8244*	,9958	,000	-10,945	-4,704
	V2B2(-)	V1C	14,3867*	,9958	,000	11,266	17,507
		V1B1(-)	10,3411*	,9958	,000	7,221	13,461
		V1B1(+)	12,3000*	,9958	,000	9,180	15,420
		V1B2(-)	7,1000*	,9958	,000	3,980	10,220
		V2C	7,7367*	,9958	,000	4,616	10,857
		V2B1(-)	3,7867*	,9958	,007	,666	6,907
		V2B1(+)	7,8244*	,9958	,000	4,704	10,945
Flag Leaf Lenght (cm)	V1C	V1B1(-)	1,1622	,8474	,867	-1,493	3,818
		V1B1(+)	-,9633	,8474	,946	-3,619	1,692
		V1B2(-)	-1,4878	,8474	,651	-4,143	1,168
		V2C	-,2000	,8474	1,000	-2,855	2,455
		V2B1(-)	3,3878*	,8474	,004	,732	6,043
		V2B1(+)	-,7000	,8474	,991	-3,355	1,955
		V2B2(-)	-3,3500*	,8474	,005	-6,005	-,695
	V1B1(-)	V1C	-1,1622	,8474	,867	-3,818	1,493
		V1B1(+)	-2,1256	,8474	,211	-4,781	,530
		V1B2(-)	-2,6500	,8474	,051	-5,305	,005
		V2C	-1,3622	,8474	,744	-4,018	1,293
		V2B1(-)	2,2256	,8474	,166	-,430	4,881
		V2B1(+)	-1,8622	,8474	,367	-4,518	,793
		V2B2(-)	-4,5122*	,8474	,000	-7,168	-1,857
	V1B1(+)	V1C	,9633	,8474	,946	-1,692	3,619
		V1B1(-)	2,1256	,8474	,211	-,530	4,781
		V1B2(-)	-,5244	,8474	,998	-3,180	2,131
		V2C	,7633	,8474	,985	-1,892	3,419
		V2B1(-)	4,3511*	,8474	,000	1,696	7,006
		V2B1(+)	,2633	,8474	1,000	-2,392	2,919
		V2B2(-)	-2,3867	,8474	,109	-5,042	,269
	V1B2(-)	V1C	1,4878	,8474	,651	-1,168	4,143
		V1B1(-)	2,6500	,8474	,051	-,005	5,305
		V1B1(+)	,5244	,8474	,998	-2,131	3,180
		V2C	1,2878	,8474	,794	-1,368	3,943
		V2B1(-)	4,8756*	,8474	,000	2,220	7,531
		V2B1(+)	,7878	,8474	,982	-1,868	3,443
		V2B2(-)	-1,8622	,8474	,367	-4,518	,793

Multiple Comparisons

Tukey HSD

Dependent Variable	(I) Group	(J) Group	Mean Difference (I-J)	Std. Error	Sig.	95% Confidence Interval Lower Bound	95% Confidence Interval Upper Bound
	V2C	V1C	,2000	,8474	1,000	-2,455	2,855
		V1B1(-)	1,3622	,8474	,744	-1,293	4,018
		V1B1(+)	-,7633	,8474	,985	-3,419	1,892
		V1B2(-)	-1,2878	,8474	,794	-3,943	1,368
		V2B1(-)	3,5878*	,8474	,002	,932	6,243
		V2B1(+)	-,5000	,8474	,999	-3,155	2,155
		V2B2(-)	-3,1500*	,8474	,010	-5,805	-,495
	V2B1(-)	V1C	-3,3878*	,8474	,004	-6,043	-,732
		V1B1(-)	-2,2256	,8474	,166	-4,881	,430
		V1B1(+)	-4,3511*	,8474	,000	-7,006	-1,696
		V1B2(-)	-4,8756*	,8474	,000	-7,531	-2,220
		V2C	-3,5878*	,8474	,002	-6,243	-,932
		V2B1(+)	-4,0878*	,8474	,000	-6,743	-1,432
		V2B2(-)	-6,7378*	,8474	,000	-9,393	-4,082
	V2B1(+)	V1C	,7000	,8474	,991	-1,955	3,355
		V1B1(-)	1,8622	,8474	,367	-,793	4,518
		V1B1(+)	-,2633	,8474	1,000	-2,919	2,392
		V1B2(-)	-,7878	,8474	,982	-3,443	1,868
		V2C	,5000	,8474	,999	-2,155	3,155
		V2B1(-)	4,0878*	,8474	,000	1,432	6,743
		V2B2(-)	-2,6500	,8474	,051	-5,305	,005
	V2B2(-)	V1C	3,3500*	,8474	,005	,695	6,005
		V1B1(-)	4,5122*	,8474	,000	1,857	7,168
		V1B1(+)	2,3867	,8474	,109	-,269	5,042
		V1B2(-)	1,8622	,8474	,367	-,793	4,518
		V2C	3,1500*	,8474	,010	,495	5,805
		V2B1(-)	6,7378*	,8474	,000	4,082	9,393
		V2B1(+)	2,6500	,8474	,051	-,005	5,305

*. The mean difference is significant at the 0.05 level.

Homogeneous Subsets

Plant Height (cm)

Tukey HSD[a]

Group	N	Subset for alpha = 0.05					
		1	2	3	4	5	6
V1C	9	20,876					
V1B1(+)	9	22,962	22,962				
V1B1(-)	9		24,921	24,921			
V2B1(+)	9			27,438	27,438		
V2C	9			27,526	27,526		
V1B2(-)	9				28,162		
V2B1(-)	9					31,476	
V2B2(-)	9						35,262
Sig.		,429	,512	,170	,996	1,000	1,000

Means for groups in homogeneous subsets are displayed.

a. Uses Harmonic Mean Sample Size = 9,000.

Flag Leaf Lenght (cm)

Tukey HSD[a]

Group	N	Subset for alpha = 0.05		
		1	2	3
V2B1(-)	9	26,212		
V1B1(-)	9	28,438	28,438	
V1C	9		29,600	
V2C	9		29,800	
V2B1(+)	9		30,300	30,300
V1B1(+)	9		30,563	30,563
V1B2(-)	9		31,088	31,088
V2B2(-)	9			32,950
Sig.		,166	,051	,051

Means for groups in homogeneous subsets are displayed.

a. Uses Harmonic Mean Sample Size = 9,000.

Comments:
- Data for PH and FLL are normally distributed (Shapiro-Wilk Sig. > 0.05), while data for mFLW are not (Shapiro-Wilk Sig. < 0.05)
- ANOVA is not performed for mFLW
- For PH and FLL there are no outliers and variances are homogeneous (Levene Sig. > 0.05).
