# Supplementary material for: The Impact of Soil-Applied Biochars From Different Vegetal Feedstocks on Durum Wheat Plant Performance and Rhizospheric Bacterial Microbiota in Low Metal-Contaminated Soil
Source: Front Microbiol. 2019 Dec 10;10:2694. doi: 10.3389/fmicb.2019.02694 (PMC6916200; doi:10.3389/fmicb.2019.02694)
Supplement: Supplementary file 1 [file Data_Sheet_1.zip › Supplementary_Material_9_Latini_et_al.docx]

Supplementary Material 9

**Table S5.** Fitting linear model: log_ab ~ Persistence

|  | Estimate | Std. Error | t value | Pr(>│t│) |
| --- | --- | --- | --- | --- |
| (Intercept) | -0.5048 | 0.006843 | -73.78 | 0 |
| Persistence | 0.07855 | 0.0007186 | 109.3 | 0 |
|  |  |  |  |  |

**Table S6.** **Analysis of variance model**

|  | Df | Sum Sq | Mean Sq | F value | Pr(>F) |  |
| --- | --- | --- | --- | --- | --- | --- |
| treatment | 3 | 53168 | 17723 | 3.215 | 0.03854 |  |
| genotype | 1 | 2102 | 2102 | 0.3814 | 0.542 |  |
| treatment:genotype | 3 | 10710 | 3570 | 0.6477 | 0.5912 |  |
| Residuals | 27 | 148830 | 5512 | N.A. | N.A. |  |

**Table S7.** **Permutation test for adonis under reduced model**

|  |  |  |  |  |  |  |
| --- | --- | --- | --- | --- | --- | --- |
|  | Df | SumOfSqs | R2 | F | Pr(>F) |  |
| treatment | 3 | 0.867 | 0.1853 | 2.562 | 0.000999 |  |
| genotype | 1 | 0.269 | 0.0575 | 2.385 | 0.008991 |  |
| treatment:genotype | 3 | 0.4973 | 0.1063 | 1.47 | 0.05395 |  |
| Residual | 27 | 3.045 | 0.6509 | N.A. | N.A. |  |
| Total | 34 | 4.679 | 1 | N.A. | N.A. |  |


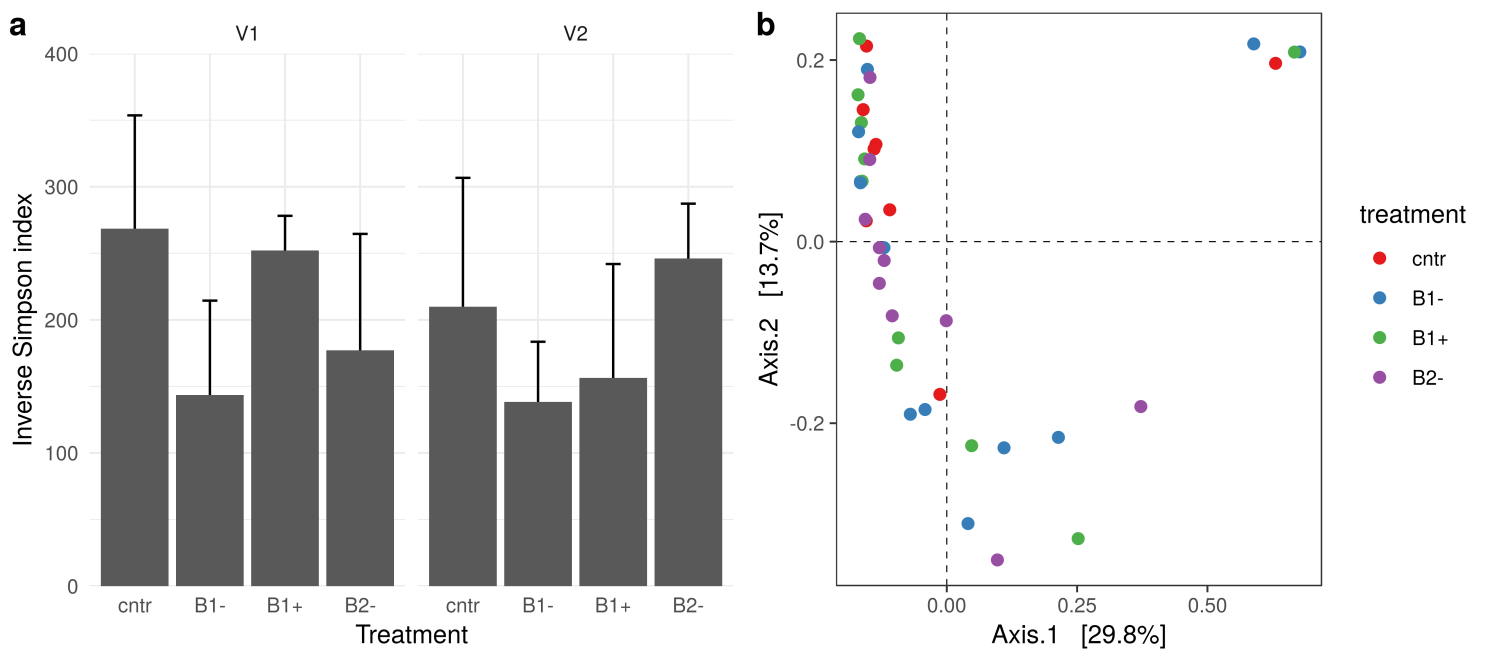


**Figure S5. a** Inverse Simpson index distribution for different treatments (C-, B1-, B1+, B2-) and genotypes (V1 and V2). **b** Principal coordinate analysis (PCoA) based on Bray-Curtis index. Different colors report different treatments.
